# Cold and Heat Stress Diversely Alter Both Cauliflower Respiration and Distinct Mitochondrial Proteins Including OXPHOS Components and Matrix Enzymes

**DOI:** 10.3390/ijms19030877

**Published:** 2018-03-16

**Authors:** Michał Rurek, Magdalena Czołpińska, Tomasz Andrzej Pawłowski, Włodzimierz Krzesiński, Tomasz Spiżewski

**Affiliations:** 1 Department of Molecular and Cellular Biology, Institute of Molecular Biology and Biotechnology, Adam Mickiewicz University, Poznań, Umultowska 89, 61-614 Poznań, Poland; magczo@amu.edu.pl; 2 Institute of Dendrology, Polish Academy of Sciences, Parkowa 5, 62-035 Kórnik, Poland; tapawlow@man.poznan.pl; 3 Department of Vegetable Crops, Poznan University of Life Sciences, Dąbrowskiego 159, 60-594 Poznań, Poland; wlodzimierz.krzesinski@up.poznan.pl (W.K.); tomasz.spizewski@up.poznan.pl (T.S.)

**Keywords:** cold stress, heat stress, stress recovery, mitochondria, proteomics, respiration, *Brassica*, angiosperms

## Abstract

Complex proteomic and physiological approaches for studying cold and heat stress responses in plant mitochondria are still limited. Variations in the mitochondrial proteome of cauliflower (*Brassica oleracea* var. *botrytis*) curds after cold and heat and after stress recovery were assayed by two-dimensional polyacrylamide gel electrophoresis (2D PAGE) in relation to mRNA abundance and respiratory parameters. Quantitative analysis of the mitochondrial proteome revealed numerous stress-affected protein spots. In cold, major downregulations in the level of photorespiratory enzymes, porine isoforms, oxidative phosphorylation (OXPHOS) and some low-abundant proteins were observed. In contrast, carbohydrate metabolism enzymes, heat-shock proteins, translation, protein import, and OXPHOS components were involved in heat response and recovery. Several transcriptomic and metabolic regulation mechanisms are also suggested. Cauliflower plants appeared less susceptible to heat; closed stomata in heat stress resulted in moderate photosynthetic, but only minor respiratory impairments, however, photosystem II performance was unaffected. Decreased photorespiration corresponded with proteomic alterations in cold. Our results show that cold and heat stress not only operate in diverse modes (exemplified by cold-specific accumulation of some heat shock proteins), but exert some associations at molecular and physiological levels. This implies a more complex model of action of investigated stresses on plant mitochondria.

## 1. Introduction

Abiotic stress, including excessive cold or heat, cause failure in the cultivation of many plant species. Such conditions may significantly reduce the yield of most major crops. Plants have various physiological and metabolic response mechanisms, which act within the complex network to avoid harm due to unfavorable environmental stimuli [1,2,3]. Understanding these mechanisms improves our knowledge of stress resistance and will allow the breeding of more appropriate plant varieties.

Numerous aspects of plant responses to cold and heat have been studied. They may differ between plant species [4,5,6]. Both low and high temperatures can decrease chlorophyll biosynthesis, significantly impeding chloroplast development and potentially resulting in photosystem II (PSII) damage [7,8,9,10,11]. Cold-grown plants generate a vast number of reactive oxygen species (ROS) [12]. Armstrong et al. [13] analyzed temperature-dependent sensitivity of leaf respiration in Arabidopsis during cold acclimation and suggested the importance of an alternative oxidation pathway in this process. Moreover, Talts et al. [14] observed that cold-treated plants often display higher rates of respiration. However, heat stress (depending on its intensity and duration) can exert particularly diverse effects on the photosynthetic apparatus [15], including increased cyclic electron flow around PSI [9,16,17,18,19].

Despite reports concerning evident alterations in plant physiological parameters during stress response, data on the correlation of those changes with mitochondrial proteomes are quite limited. Organellar proteomic analyses, including mitochondrial ones, may help to reveal the intrinsic mechanisms of stress response by elucidating the relationship between protein variations and general plant tolerance to environmental factors [20]. Nowadays, characterization of total proteomes or sub-proteomes of important crop and vegetable plants, including cauliflower (*Brassica oleracea* var. *botrytis*), appears to be very important [21,22,23,24,25,26].

The plant mitochondrial proteome is a very dynamic entity which can be remodeled in a plethora of environmental conditions and developmental signals [27,28]. It is known that dozens of nuclear genes encoding mitochondrial proteins respond to stress conditions form a functional network [29]. Using an integrative approach, Cui et al. [30] found 503 Arabidopsis mitochondrial proteins participating in a stress protein interaction network. This suggests the general dependence of plant mitochondria on other plant cell compartments during stress response. Furthermore, Taylor et al. [31] estimated that only 22% of total Arabidopsis organellar proteins that are stress-responsive comprise mitochondrial proteins. It seems that the number of mitochondrial proteins involved in stress response is still underestimated, due to limited complexity of some reports and the fact that a significant number of results came from analyses of total plant proteomes and main metabolic pathways only [25,32,33,34]. It should be mentioned that the number of low-abundant mitochondrial proteins responsive to temperature stress is still far from being understood [31]. Recently, these issues were improved by the application of isobaric tags for the absolute quantification (iTRAQ) or label-free peptide counting coupled with liquid chromatography-tandem mass spectrometry (LC-MS/MS) [35,36,37,38]. Using a gel-free approach, Tan et al. [39] found that cold stress led to a concerted decrease in respiratory protein level, accompanied by an increase in abundance of some import/export protein machinery components. However, the overall amount of cold-responsive proteins was smaller, when compared to other suboptimal stimuli.

Although it is known that temperature stress modulates mitochondrial protein activity, level, biogenesis and interactions [40,41,42], crucial steps of achieving appropriate coordination during mitochondrial biogenesis in stress need to be further investigated. For instance, Giegé et al. [43] showed that regulation of mitochondrial biogenesis in Arabidopsis cell cultures during sugar starvation seems to be rather coordinated at the complex assembly. Approaches linking molecular and physiological data dealing with temperature stress impact on mitochondria are still welcomed. Some mitochondrial proteins (e.g., alternative oxidase [AOX]) are ‘classical’ modulators of stress response among plants [44,45,46]. Regulation of diverse *AOX* genes varies between monocots and dicots. In a number of plant species, the alterations of AOX protein are less pronounced [47,48]. In addition, AOX may not to be increased in abundance by certain stress treatments, for example chilling [49,50]. The latter phenomenon was also confirmed in our previous study [41]; we reported a significant decline in AOX level in cauliflower mitochondria under cold stress and recovery. Overall *AOX* gene family responses on proteomic and transcriptomic levels were only partially associated and AOX was a suggested target of translational regulation in diverse temperature treatments. In tobacco (*Nicotiana tabacum*) leaves, abundance of this protein reached a maximum after 48 h of cold stress and slowly decreased afterwards [51]. This highlights the importance of the length of cold stress treatment for the plant to gain acclimation, presumably by the induction of regular changes in the transcriptome first [52].

Assuming limitations of the deposited data, this work was undertaken to gain a comprehensive view about the influence of cold and heat treatment (as well as cold and heat recovery) on the cauliflower mitochondrial proteome in relation to leaf transpiration and respiration rate, stomatal conductance, rate of leaf photosynthesis, photorespiration as well as chlorophyll content and fluorescence. The current study extends our previous complexomic and functional data [41]. To determine mitochondrial proteome response in relation to plant respiration, we aimed to (1) investigate the dynamic nature of the mitochondrial proteome under cold and heat treatment and stress recovery; (2) identify the most variable proteins in cauliflower inflorescence mitochondrial extracts; and (3) link proteomic and discussed metabolic/functional aspects with alterations of analyzed physiological parameters. On the whole, the broader set of identified proteins responding to cold/heat stress and after stress recovery, which correlate with alterations in plant respiration and some general metabolic demands, was able to be characterized in cauliflower mitochondria.

## 2. Results

### 2.1. Proteome Maps of Cauliflower Mitochondria under Stress Conditions

**Figure 1:**
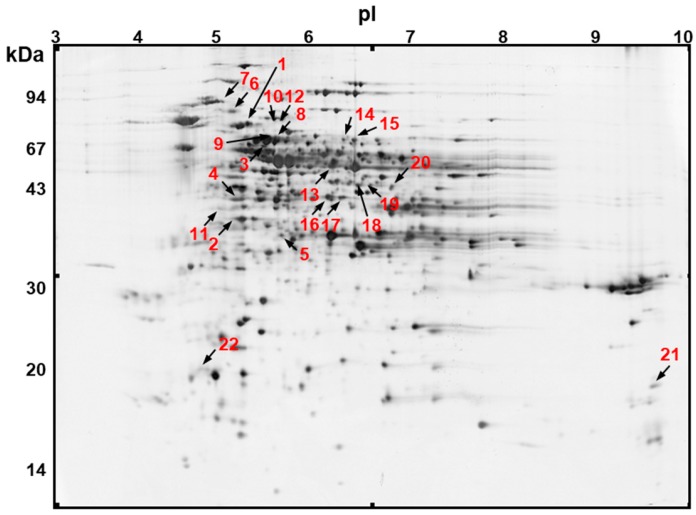
Position of varying spots on 2D silver-stained master gel of cauliflower curd mitochondrial proteome (in total 100 µg of mitochondrial proteins pooled from all experimental variants), including 694 repeatable spots. 24-cm immobilised pH gradient strips (linear pH 3–10) for the first dimension and precast Ettan DALT 12.5% SDS-polyacrylamide gels for the second dimension were used. It shows the position of the 22 variable spots that were mapped and identified (they appear also in Figure 1, Figure S1 and S2 and Table 1). Protein molecular mass standards (Thermo Scientific, Gdańsk, Polska) sizes are given in kilodaltons (kDa); pI-isoelectric point. Further experimental details in Materials and Methods.

Mitochondrial proteins isolated from curds of control plants and from plants submitted to cold or heat treatment were resolved by two-dimensional gel electrophoresis (2D PAGE). We also examined the mitochondrial proteomes from curds of stress-recovered cauliflower plants with the idea to study the impact of stress on the mitochondrial proteome under stress recovery (Figure S1). 2D gels for investigated variants, including control, were run in triplicate. Individual gel replicates are shown in Figure S2. In order to create master gel, we chose the image of control variant as the reference and then we added the specific spots detected on the gels of remaining variants. The number of spots on silver-stained 2D gels varied from 347 to 511 between all analyzed variants, including the control one. Thus, 694 different spots were taken into account for the building of a synthetic silver-stained master gel (Figure 1). Contrary to silver-stained gels, the number of protein spots on colloidal Coomassie Brilliant Blue (CBB)-stained 2D gels was lower. Finally, for the analysis of spot variation, only 413 spots representing highly abundant proteins from silver-stained gels were taken into account.

### 2.2. Identification of Variable Protein Spots

Twenty two spots (3.2% on the silver-stained master gel) were significantly variable (verified by the analysis of variance [ANOVA] and Tukey’s honest significant difference [HSD] test) as detected using Image Master 7 Platinum software. Spot positions depicted in Figure 1, Figure S1 and S2 were calculated from three biological replicates. All spots were successfully identified by LC-MS/MS. The obtained data were used for searching Mascot against the National Centre for Biotechnology Information (NCBI) database (version 20100203). Cauliflower stress-responsive mitochondrial proteins were identified by using the *Viridiplantae* section of the database (with the aid of Arabidopsis and *B. oleracea* nuclear and mitochondrial [53] genomes). To avoid possible misidentifications resulting from large datasets, as pointed out by Schmidt et al. [24], we were able to set the false positive rate threshold to 5%. Identifications of protein spots are presented in Table 1 and properties of individual peptides for each protein spot are given in Table S1. As illustrated, all 22 spots represented 16 non-redundant stress-responsive proteins. We did not exclude spots with multiple protein identification from the analyses, however, we focused on protein identifications (for all stress-responsive spots) confirmed by sufficient parameter quality (highest MOWSE score, emPAI, peptide number and coverage). The disproportion between the quality of mentioned parameters for the initial and remaining records in the MASCOT search list allowed us to do so. The percentage of sequence coverage ranged from 14% to 42% and the total number of identified peptides varied from 12 to 301. Among all spots, mitochondrial proteins in all but one spot (spot No. 13 corresponding to *Brassica napus* protein sequence) were identified based on their high similarity to Arabidopsis sequences. In addition, 17 Arabidopsis proteins also showed a very high or 100% sequence identity with *B. oleracea* var. *oleracea* records (Table S2). The experimental molecular mass corresponded roughly to the theoretical value for the majority of spots. Some proteins including phosphoglycerate kinase isoform 1 (PGK1; spots No. 4, 10; Table 1), mitochondrial elongation factor Tu (mtEF-Tu; spots No. 16, 17), isocitrate dehydrogenase (IDH; spot No. 18) and citrate synthase (CS; spots No. 19, 20) showed a few kDa decrease in molecular mass between theoretical and gel values. We are rather convinced that this did not result from the excessive proteolysis in cauliflower mitochondria.

Seven spots (approximately 1% of all) displayed significant variations in their abundance after cold stress and cold recovery. According to Tukey’s HSD test, four proteins, including three members of the heat shock protein (HSP) family (spots No. 1, 6, 7) and 3-phosphoglycerate dehydrogenase (PGDH)–like protein (spot No. 3) were increased in abundance during cold treatment. After cold recovery another three proteins were significantly increased in abundance, namely pyruvate dehydrogenase subunit β (PDHβ; spot No. 2), PGK1 (spot No. 4) as well as NAD^+^-dependent malate dehydrogenase (MDH; spot No. 5; Table 1).

After heat stress and heat recovery, 15 responsive spots (representing 11 non-redundant proteins) were identified (approximately 2.2% of all spots; Table 1). Four proteins PGDH-like protein, Δ-1-pyrroline-5-carboxylate dehydrogenase (P5CDH), mtEF-Tu as well as CS were represented by double spots (No. 8/9, 14/15, 16/17 and 19/20, respectively) displaying slightly different molecular mass and pI values. According to Tukey’s HSD test, it appeared that three proteins significantly raised their level during heat treatment: PGDH–like protein (spots No. 8), mtEF-Tu (spots No. 17), as well as mitochondrial ATP synthase subunit d (ATPQ; spot No. 22), but two proteins: P5CDH (spot No. 15) and CS (spots No. 19/20) were decreased in abundance.

**Figure 2:**
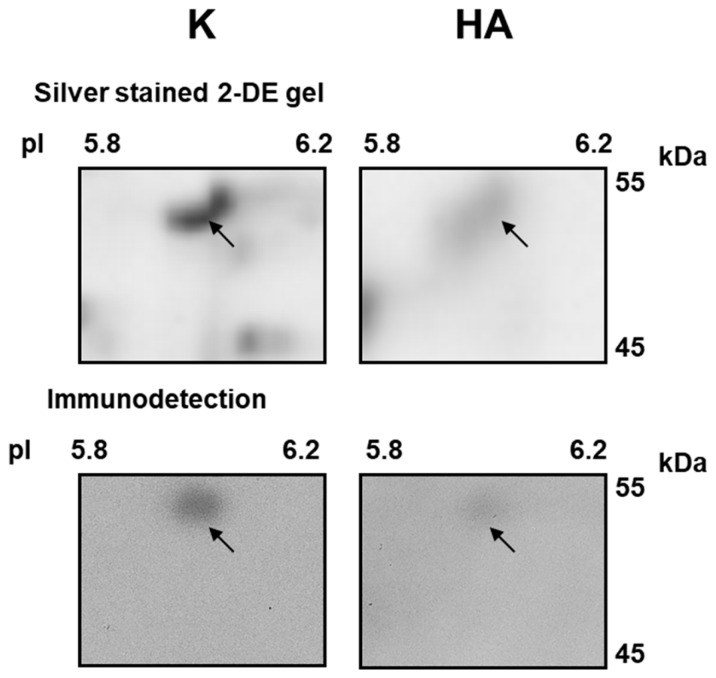
Immunoblotting of ATP1 on 2D blots containing cauliflower curd mitochondrial proteins from control grown plants (**K**) and from heat-recovered plants (**HA**). 100 μg of mitochondrial proteins were loaded onto all gels. 24-cm immobilized pH gradient strips (linear pH 3–10) for the first dimension and precast Ettan DALT 12.5% SDS-polyacrylamide gels for the second dimension were used. For protein transfer onto Immobilone membrane, a semidry system was applied. Blots were probed with polyclonal antibodies raised against mitochondrial ATP synthase subunit α (ATP1). Detection was carried with chemiluminescence assays after incubation with HRP-conjugated secondary antibody. Representative results (from triplicates) are shown. For the comparison, panels showing fragments of silver-stained 2D gels that contains spots for ATP1 are displayed. Arrows (indicated in each blot) show the position of ATP1 on immunoblots and 2D gels. Protein molecular mass standard (Thermo Scientific, Gdańsk, Poland) sizes are given in kilodaltons (kDa); pI-isoelectric point. Further experimental details in the text.

Some variations were also observed after heat recovery. Here, we detected a more intense accumulation of a broad set of proteins, namely PGDH-like protein (spots No. 8, 9), PGK1 (spot No. 10, succinyl-CoA ligase subunit β (SCLβ; spot No. 11), chaperonin 10 (CPN10; spot No. 21), and ATPQ (spot No. 22). Notably, CPN10 extensively increased in abundance. In those conditions, we also noticed a significant decline of the ATP synthase subunit α (ATP1; spot No. 13), mitochondrial processing peptidase subunit β (MPPβ; spot No. 12), P5CDH (spots No. 14, 15), mtEF-Tu (spots No. 16/17), IDH (spot No. 18) and CS level (spots No. 19/20; Table 1). In addition, with the help of polyclonal antibodies, we verified the abundance of ATP1 (the only protein from our data encoded by the mitochondrial genome) after heat recovery on 2D immunoblots. As illustrated in Figure 2 (all 2D immunoblot replicates are shown in Figure S2), the respective variations of this protein assayed by immunoblotting roughly followed the protein variations on the silver stained 2D gels. Notably, four proteins that were identified as double spots showed very similar response after heat and heat recovery, which is in favour for the correctness of their assignments (Table 1).

Full peptide data from the error-tolerant MASCOT search (Table S3a) allow to get a general view on the extent posttranslational protein modifications (PTMs) within proteins (listed in Table 1) from double spots (No. 8/9, 14/15, 16/17 and 19/20). As was shown, different proteins were identified in each spot pair. Protein multi-spotting frequently accompanies 2D gel data, however, we only investigated stress-responsive double spots (Table 1). We wanted to check whether the presence of unknown PTMs could be associated with protein multi-spotting (Table S3b). We estimated a number of modified residues from spectra of tryptic peptides as well as expected molecular mass difference from the extent of given PTM between compared spots. We focused on phosphorylations, deamidations, methylations, formylations and ethylations. Because no phosphoprotein enrichment was performed, we were unable to further characterize the phosphoproteome in cauliflower mitochondria, despite phosphorylated residues accounting mostly for total theoretical molecular mass difference between double spots. However, only limited correlation was found between this value and the experimental molecular mass difference for double spots. Therefore, multi-spotting of some analysed proteins came rather from non-investigated modifications and/or from the expression of gene family members.

### 2.3. Functional Categorization of Identified Proteins

**Figure 3:**
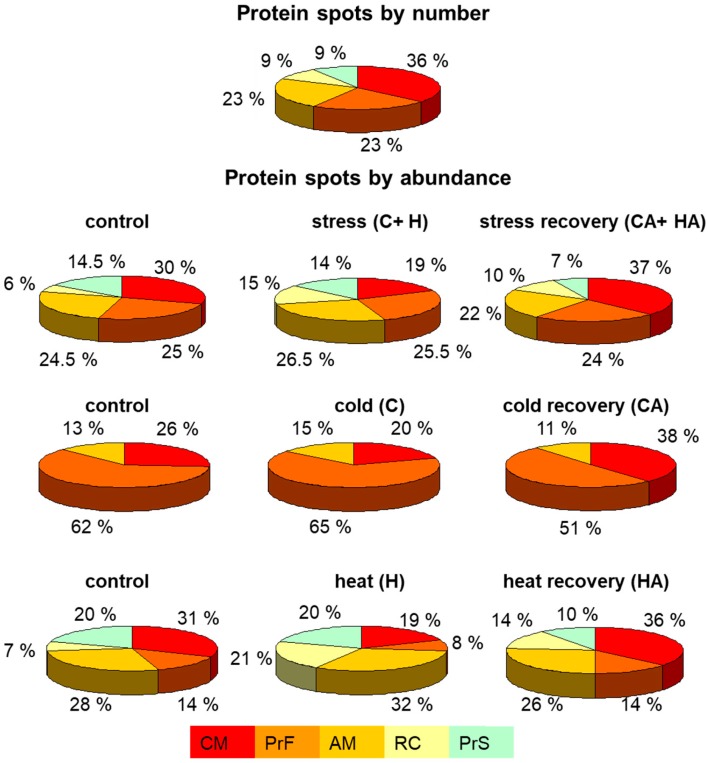
Functional categorization of cauliflower stress-responsive protein spots, analysed by 2D PAGE. Spots were analysed both according to their number (**upper**) as well as densitometrical volume (abundance; **below**) calculated for each of functional groups in control grown plants, cold- or heat-stressed plants (**C** or **H**, respectively) and cold- or heat-recovered plants (**CA** or **HA**, respectively). Bar legend of categories: **CM**—carbohydrate metabolism, **PrF**—protein fate, **AM**—amino acid metabolism, **RC**—respiration (respiratory chain components), **PrS**—protein synthesis.

Based on Arabidopsis protein orthologues, we used a functional categorization (FunCat) scheme at the Munich Information Center for Protein Sequences database (Available online: http://ibis.helmholtz-muenchen.de/funcatDB/) for clustering of stress-responsive proteins resolved on 2D gels into five functional categories (Figure 3 and Table 1).

Counting the number of spots within each category (Figure 3, panel: protein spots by number), it appeared that the majority of cauliflower mitochondrial protein spots responsive to cold and heat stress belonged to the class participating in carbohydrate metabolism, including tricarboxylic acid (TCA) cycle components (about 36% spots) as well as amino acid metabolism and protein fate (each of approximately 23%). The next ones were represented by respiratory chain (RC) components and protein synthesis apparatus (each of approximately 9%). Interestingly, eight spots (36%) representing six proteins were already annotated as stress responsive in the MIPS database.

Spots linked to RC components increased in abundance after heat stress, as well as after heat recovery, however, the ones linked to amino acid metabolism were upregulated after cold and heat stress. In contrast, spots linked with carbohydrate metabolism decreased in abundance after cold and heat, but markedly upregulated in cold- and heat-recovered plants (Figure 3, panel: protein spots by abundance), Interestingly, the total abundance of spots related to the protein fate showed some increase after cold, but neither after heat stress (where it was decreased), nor after recovery phase. It seems that the majority of identified protein spots that belonged to the protein fate class appeared responsive in cold stress and cold recovery, which indicates its overall importance in low temperature response in cauliflower mitochondria. Many protein functional classes, however, were regulated by heat and heat recovery (Figure 3, panel: protein spots by abundance, at the bottom).

### 2.4. Effect of Cold Stress on Abundance of Additional Mitochondrial Proteins

**Figure 4:**
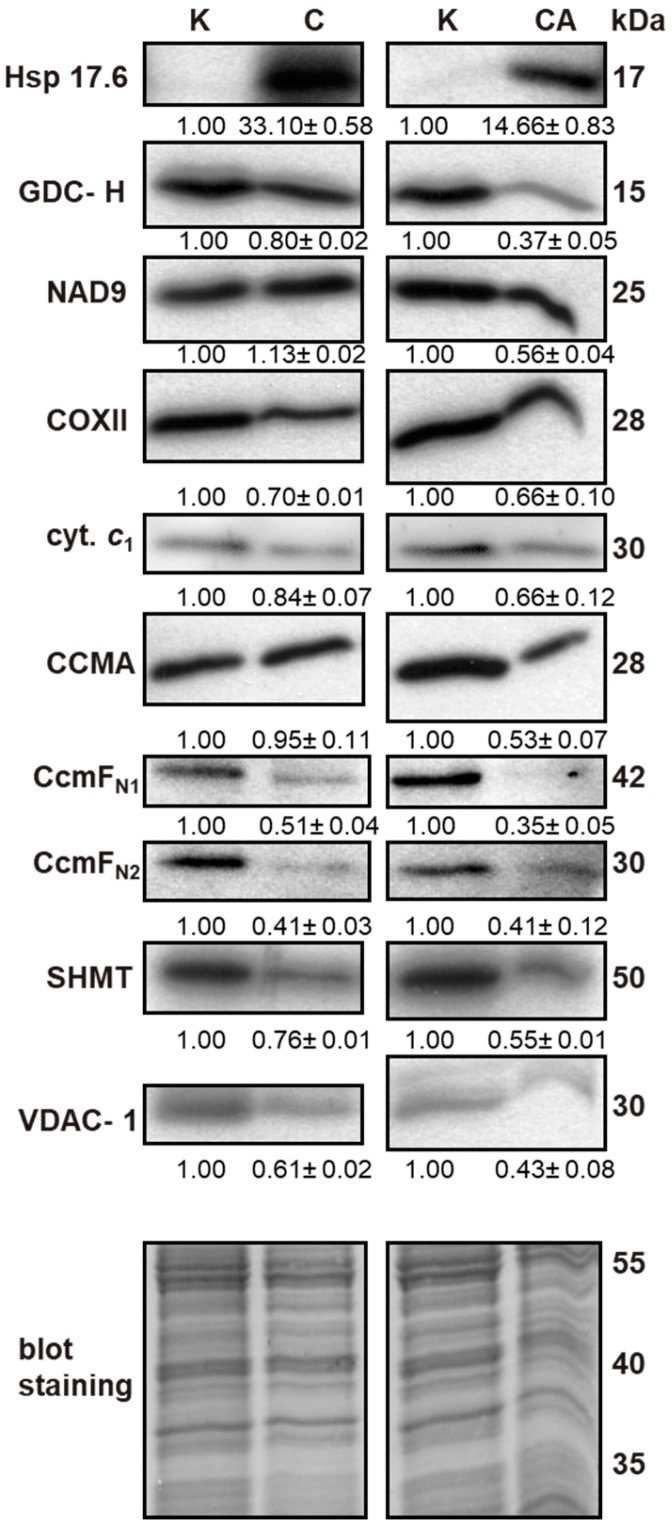
Immunoblotting of proteins from control grown plants (**K**), cold-stressed plants (**C**) and from cold-recovered plants (**CA**). About 10 μg of mitochondrial proteins from cauliflower curds were loaded onto SDS-polyacrylamide gels. Proteins were transferred onto an Immobilone membrane using a semidry system. All assays were performed using specific primary antibodies raised against heat shock protein 17.6C class I (Hsp17.6), glycine decarboxylase subunit H (GDC-H), NADH dehydrogenase (CI) subunit 9 (NAD9), cyt. *c* oxidase (CIV) subunit 2 (COXII), cyt. *c*_1_, cyt. *c* maturation proteins (CCMA, CcmF_N1_, CcmF_N2_), serine hydroxymethyltransferase 1 (SHMT) and voltage-dependent anion channel 1 (VDAC-1). Detection was carried out with chemiluminescence assays after incubation with HRP-conjugated secondary antibody. Representative results from triplicates are shown. The relative abundance of bands is given below each panel. The abundance in stress conditions (value ± SD) is standardized to 1.00 in control variants. For the loading control, blot staining with Coomassie Brilliant Blue is additionally shown. Protein molecular mass standard (Thermo Scientific, Gdańsk, Poland) sizes are given in kilodaltons (kDa). Further experimental details in Materials and Methods.

Due to the fact that the number of cold-regulated proteins was lower than those regulated by heat stress in cauliflower mitochondria, we decided to verify our analyses by additional immunoblotting assays (Figure 4). All immunoblot replicates are shown in Figure S2. The level of selected proteins was monitored in mitochondria isolated from curds of cauliflower plants grown either in control conditions or submitted to cold, or from cold-recovered plants. To verify protein loading, blots were Coomassie-stained.

We assayed the level of glycine decarboxylase subunit-H (GDC-H), serine hydroxymethyltransferase 1 (SHMT), mitochondrial porine isoform 1 (VDAC-1), some OXPHOS proteins, including complex I (CI) subunit 9 (NAD9), cytochrome *c*_1_, complex IV (CIV) subunit 2 (COXII) as well as proteins engaged in cytochrome *c* (cyt. *c*) maturation in plant mitochondria, particularly ABC transporter I family member 1 (CCMA) and CcmF N-terminal-like mitochondrial proteins 1 and 2 (CcmF_N1_ and CcmF_N2_, respectively). With the application of specific antibodies, we also investigated the level of cytoplasmic small Hsp17.6 of class I (sHsp17.6C-CI), that interacts with mitochondria under temperature stress [54]. It appeared, that the level of Hsp17.6C-CI associated with mitochondrial membranes increased extensively after cold stress and remained quite high after cold recovery (Figure 4). The abundance of GDC-H showed almost a three-fold change decrease after cold recovery, but only slightly after cold stress. A similar decrease in the level of CcmF_N1_ and CcmF_N2_ proteins was observed in cold and cold recovery conditions (up to two- and three-fold change, respectively). In contrast, the accumulation of the CCMA transporter protein was not affected by cold; however, it was decreased (by almost 50%) after cold recovery. In the tested conditions, the relative abundance of SHMT and VDAC-1 was also decreased. Regarding RC proteins, we detected a small upregulation of NAD9 subunit of CI after cold stress and a subsequent major decline under cold recovery as well as a small downregulation of COXII and cyt. *c*_1_ in stress (Figure 4).

### 2.5. Association between Protein and Transcript Level

Besides analyses of cauliflower mitochondrial proteome, we studied how proteomic response is accompanied by transcript alterations. For the rapid assessment of both patterns, we employed RT-semiqPCR and the level of five mitochondrial and 11 nuclear messengers was assayed (Figure S3) and compared with the abundance of some OXPHOS proteins encoded by mitochondrial genome and proteins coded by nuclear genome investigated in this study (Section 2.2 and Section 2.4).

None of the mitochondrial mRNAs showed alterations in their abundance associated with the protein level. *nad9* and *coxII* messengers (coding for CI subunit 9 and CIV subunit 2) were regulated inversely compared with the respective proteomic data in cold and cold recovery. *atp1* transcripts (coding for ATP synthase subunit 1) responded only in cold recovery. Furthermore, expression profiles of genes coding for subunits of the same protein complexes (e.g., *nad* genes for CI and *atp*/*ATP* genes for ATP synthase) were largely unassociated. This is true both for selected mitochondrial as well as nuclear genes. The level of *ATP2* nuclear transcripts was slightly decreased in cold and heat, and decreased furthermore in stress recovery.

Parallel RNA/protein accumulation patterns were noted in case of the only three nuclear genes coding for HSP70 isoform 1 (*HSP70-1*) in cold and cold recovery, mitochondrial processing peptidase subunit (*MPP*β) as well as for Δ-1-pyrroline-5-carboxylate dehydrogenase (*P5CDH*) in heat recovery. However, those variations were quite minor. In case of the remaining regulations, we can see that the decreased mRNA level (particularly for some transcripts, e.g., *Hsp17.6* in cold and cold recovery and *SCL*β together with *CPN10* in heat recovery) did not correlate with the increased protein abundance in stress and recovery. Our results indicate also for the down-regulation of transcripts coding for two enzymes of Pro catabolism: proline dehydrogenase (*PRODH*) and Δ-1-pyrroline-5-carboxylate dehydrogenase (*PRODH* and *P5CDH*, respectively) in cold and heat recovery (Figure S3).

### 2.6. Cauliflower Physiological Responses to Cold and Heat Stress, and after Stress Recovery

**Figure 5:**
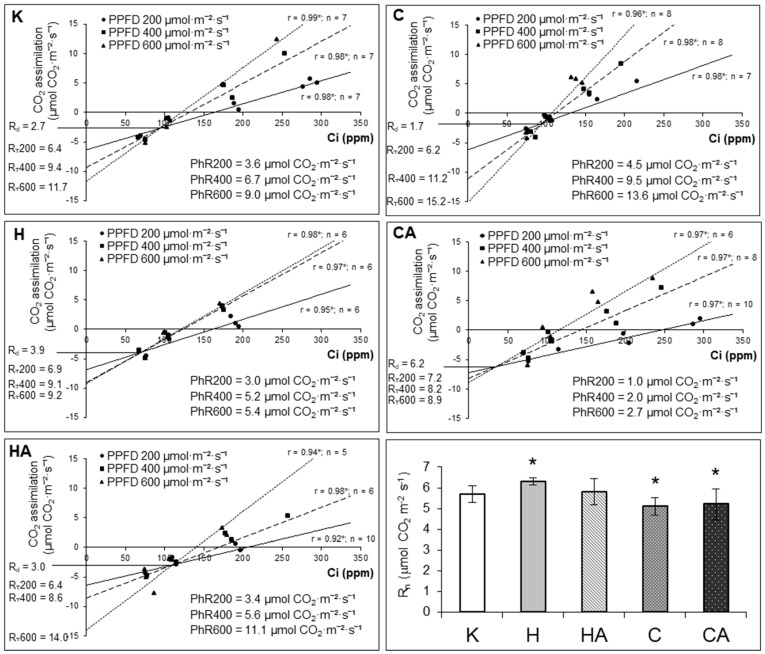
Changes in cauliflower leaf light (R_d_), dark (R_n_) respiration as well as total light (R_T_) respiration and (PhR) photorespiration (all expressed in µmol CO_2_·m^−2^·s^−1^) at 200 (R_T_200 and PhR200), 400 (R_T_400 and PhR400) and 600 (R_T_600 and PhR600) µmol·m^−2^·s^−1^ illumination rate in control grown (**K**), heat-stressed (**H**), heat-recovered (**HA**), cold-stressed (**C**) and cold-recovered (**CA**) plants. All parameters were measured on 3-month-old plants with fully developed leaves with the application of an infrared gas analyser. Data were recorded after at least 2 h of illumination. During the experiment, each of the analysed leaves were placed into a 6-cm^2^ chamber of the analyser. Results were recorded after initial leaf acclimation to the desired light and CO_2_ concentration, relative humidity and temperature. The R_d_ rate was determined according to the Laisk [55] method. The photorespiration rate for each PPFD value was determined as the difference between R_T_ and R_d_ values. Error bars denote ± S.D. Asterisks indicate significantly different curves at *p* = 0.05 (Student’s *t*-test). Further experimental details in Materials and Methods.

We also studied how leaf respiration was affected after cessation of cold and heat treatment as well as after post-stress plant recovery. By using an appropriate assay [55], we determined mitochondrial respiration in the light (non-photorespiratory intracellular decarboxylation; R_d_) in gas phase as the rate of CO_2_ release. In addition, we also measured the rate of respiration of darkened leaves (R_n_). It appeared, that the respiratory production of CO_2_ in illuminated leaves was lowered in cold-stressed plants; however, under cold recovery, a significant burst of R_d_ was observed. R_n_ rate was also lowered after cold treatment and remained so after cold recovery. In contrast, both R_d_ and R_n_ rates significantly increased in heat stress and decreased almost to control stage values after heat recovery (Figure 5).

**Figure 6:**
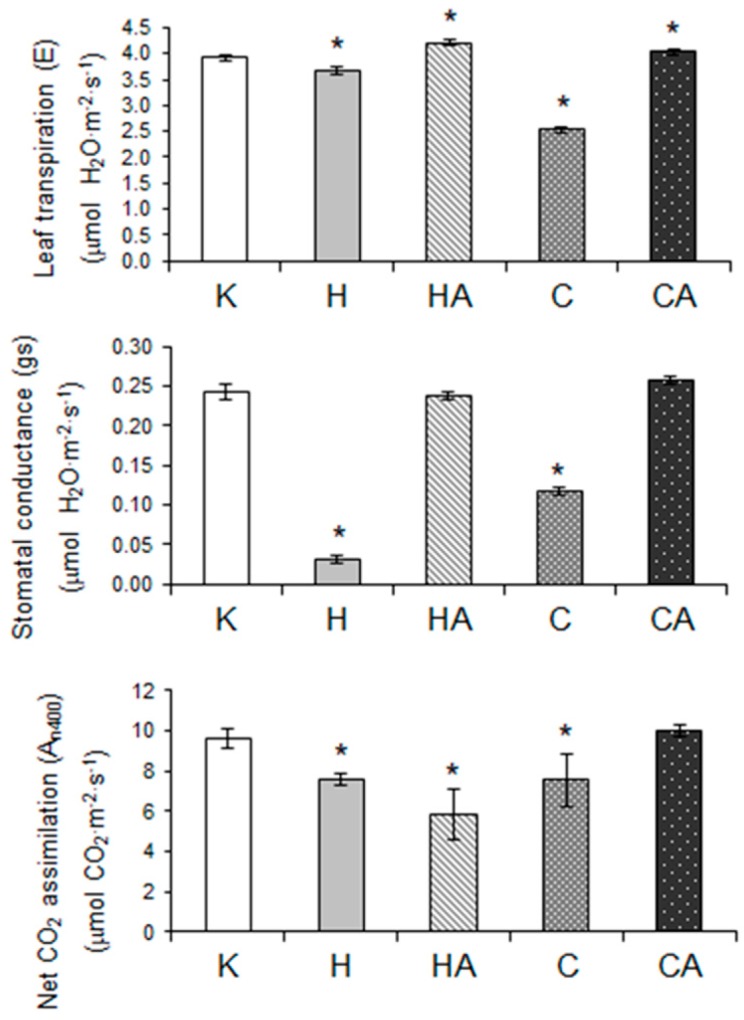
Changes in cauliflower leaf transpiration, stomatal conductance to water vapour and net CO_2_ assimilation rate at 400 μmol·m^−2^·s^−1^ illumination in control grown (**K**), cold-stressed (**C**), heat-stressed (**H**), cold-recovered (**CA**) and heat-recovered (**HA**) plants. All parameters were measured on 3-month-old plants with fully developed leaves with the application of an infrared gas analyser. Data were recorded after at least 2 h of illumination. During the experiment, each of the analysed leaves were placed into a 6-cm^2^ chamber of the analyser. Results were recorded after initial leaf acclimation to the desired light and CO_2_ concentration, relative humidity and temperature. Bars are means ± SD (*n* > 3) and asterisks indicate significant differences (*p* < 0.05; Student’s *t*-test) from the control (**K**). Further experimental details in Materials and Methods.

To gain a more complete view of cauliflower plant physiological status, we also assayed the impact of stress conditions and stress recovery on leaf transpiration (E) rate, stomatal conductance (g_s_) as well as essential photosynthetic parameters. We detected a decrease in E rate as well as lower g_s_ value under cold and heat stress, but not after cold recovery. However, after heat recovery, leaf transpiration was slightly elevated (Figure 6).

To investigate whether all those responses were also accompanied by impaired photosynthetic performance, we also measured the rate of net CO_2_ assimilation at three photosynthetic photon flux densities (PPFDs)—200, 400 and 600 µmol·m^−2^·s^−1^. Here, the net photosynthesis intensity was presented only for 400 µmol·m^−2^·s^−1^ (A_n400_), which appeared the most optimal PPFD; the respective net CO_2_ assimilation rate values at the remaining photon flux densities (A_n200_, A_n600_) followed similar to A_n400_ trends in stress response. The rate of A_n400_ was markedly decreased after cold, heat and also after heat recovery and, generally, it accompanied similar variations in stomatal closure and leaf transpiration (Figure 6).

**Figure 7:**
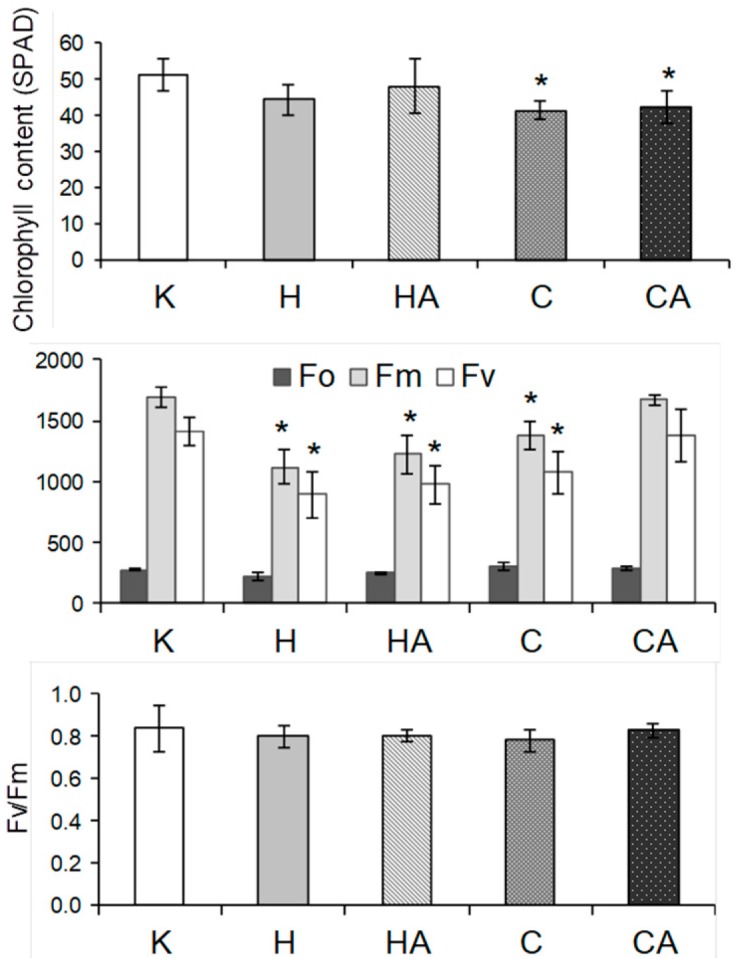
Changes in cauliflower leaf chlorophyll content minimal (Fo), maximal (Fm) and variable (Fv) fluorescence and Fv/Fm ratio in control grown (**K**), cold-stressed (**C**), heat-stressed (**H**), cold-recovered (**CA**) and heat-recovered (**HA**) plants. Chlorophyll content measured with a chlorophyll meter was expressed in relative units. Chlorophyll fluorescence was measured using a portable fluorometer. Before measurement, leaves were dark adapted for 30 min. Photochemical efficiency of PSII could be estimated from the Fv/Fm ratio, where Fv is the difference between Fm and Fo. Bars are means ± SD (*n* > 3) and asterisks indicate significant differences (*p* < 0.05; Student’s *t*-test) from the control (**K**). Further experimental details in ‘Materials and Methods’.

Notably, all those parameters did not correlate with alterations in variable (Fv) to maximal (Fm) chlorophyll fluorescence ratio, which appeared relatively constant for all investigated stress conditions. However, Fv and Fm significantly decreased both after heat and cold stress as well as after heat recovery. The relative chlorophyll content (assayed by chlorophyll meter) was affected only after cold stress and cold recovery (Figure 7). Due to the fact that in cauliflower curds, which are not involved in CO_2_ assimilation, the decrease in abundance of two main photorespiratory enzymes (GDC and SHMT) was noticed (Figure 4), we also aimed to investigate photorespiration (PhR) in photosynthetically active organs in fully expanded leaves. Using Laisk’s [55] method, we determined the ratio of photosynthetic rate under three investigated PPFDs between ambient and low CO_2_ concentration. It appeared that PhR at all PPFDs markedly increased in cold-stressed plants; however, after cold recovery it was severely impaired. In contrast, heat stress and heat recovery resulted only in the slight decline of PhR200 and PhR400 values, whereas PhR600 was more affected at heat stress, but it was recovered after heat recovery (Figure 5).

Overall, we showed that cauliflower plants, besides mitochondrial proteome plasticity at the physiological level, display only partial but diverse alterations in various photosynthetic and respiratory parameters.

## 3. Discussion

### 3.1. Identification of Cauliflower Stress-Responsive Proteins by MS Analysis

In order to obtain a more general view of the impact of cold and heat stress on the functioning of cauliflower mitochondria, we began our study by their proteome analysis. Using 2D PAGE, 22 stress-responsive spots representing 19 non-redundant proteins were selected. Although some proteins belong to the general components of the abiotic stress response [40], in this study the list of cauliflower mitochondrial proteins responsive to temperature stress was broadened by stress recovery data showing new candidates (Table 1). Our previous studies suggested that stress recovery is associated with the possible acquiring of stress tolerance by cauliflower displaying some alterations within the mitochondrial OXPHOS and dehydrin-like proteins [41,56]. We would like to complement the study of mitochondrial complexome [57] by extended physiological and proteomic analyses and to follow the importance of stress recovery conditions in such assays.

Cauliflower is closely genetically related with other *Brassica* species and the identification of mitochondrial proteins was conducted based on protein sequence similarity between *Brassicaceae* members. Schmidt et al. [24] and Zhu et al. [36] have identified some proteins (e.g., ATPQ, CPN10, MDH, PDHβ and HSP81-1) that appeared to be stress-responsive in our study. The presence of protein spots containing glycolytic enzymes (for instance PGK1) (Table 1) was not curious, because this enzyme was reported to be associated with outer mitochondrial membrane [58,59]. Such a finding was concluded mainly from the measurements of its enzymatic activity in mitochondrial extracts, however, the cytosolic member of this enzyme family (At1g79550), distinct to the Arabidopsis homolog (At3g12780) of cauliflower protein, was also identified in a large protein complex associated with mitochondrial membranes [58,60]. Interestingly, Arabidopsis PGK1 ortholog from plastid proteome showed cold response [61], whereas cauliflower mitochondrial protein was affected after heat recovery.

Four cauliflower mitochondrial proteins (3-PGDH, P5CDH, mtEF-Tu and CS) were represented as double spots. The presence of multiple spots on 2D gels was reported in numerous proteomic analyses, also including proteins analysed in this study (Figure S1) [21,22,32,50,62,63,64,65,66,67]. Consequently, we determined the extent to which posttranslational modifications might be responsible for the presence of multiple spots for the investigated proteins. Due to the lack of quantitative analysis including laborious enrichment of protein extracts in modified proteins and technical limitations of our protein separation methods, we were not able to accurately analyse majority of PTMs. Instead, we focused on a few selected modifications only (Tables S3a and S3b). However, various algorithms used for the PTM prediction among Arabidopsis emphasize that our data is largely novel and also significantly broadens deposited records. Among investigated modifications, many phosphorylated and methylated peptides were detected. Phosphorylation, together with oxidation, belong to the most important PTMs, regulating the activity of many stress-responsive proteins; in plant mitochondria, phosphorylation has particularly been studied in detail [34,68,69,70,71]. Energy and transport proteins, HSPs and even RC components were identified as potent phosphorylation targets [71]. Among proteins that were present in multiple spots in our study, it was shown that rice (*Oryza sativa*) CS can be phosphorylated [72] and mtEF-Tu was subjected to oxidation [73]. Overall, multi-spotting of cauliflower mitochondrial protein may depend not only on the presence of different PTMs, but largely on multigenic families coding novel protein isoforms, which resulted from the complex evolution of *Brassica* nuclear genomes as they underwent numerous chromosomal doublings, hybridizations and rearrangements [74]. More sensitive and quantitative proteomic assays should be implemented in the future for the better characterization of PTMs in cauliflower mitochondrial proteome.

We also noticed minor differences in molecular mass between nominal and observed values of some cauliflower mitochondrial proteins (Table 1). However, such discrepancies may be even more evident due to protein degradation [75]. Taylor et al. [50] and Imin et al. [76] have shown that abiotic stress could induce accumulation of protein degradation products. We routinely used protease inhibitors for the preparation of mitochondria, therefore, we think that extensive proteolysis could not account for major molecular mass discrepancies. Overall, despite the general similarity of 2D maps, it seems that numerous mitochondrial proteins may slightly differ in some physicochemical properties between Arabidopsis and cauliflower. We expected this from our previous analyses [23].

### 3.2. Variations in Pattern of Cauliflower Mitochondrial Proteome in Stress and Stress Recovery, and Their Metabolic Relevance

From the identified 16 stress-responsive proteins, at least an ca. two-fold change in variations in protein abundance were shown for most of them (Table 1). Under heat stress and heat recovery more proteins which varied in abundance were identified, compared to cold/cold recovery. From our data, only four stress-responsive proteins (ATP1, NAD9, COXII, CcmF_NI_, CcmF_NII_) are encoded in the plant mitochondrial genome (Table 1, Figure 4). Such a discrepancy may be due to the fact that the proteomic data allow the estimation of only a limited amount of mitochondrial proteins participating in various stress responses [31], therefore, literature inventories of those proteins are still far from complete. Rurek [40] lists almost 82 cold- and 52 heat-responsive plant mitochondrial proteins and only five proteins encoded by mitochondrial genome within them. In the up-dated Heidarvand et al. [42] review, only four proteins encoded in mitochondria contrast with the 44 nuclear-encoded cold responsive proteins. However, the modulation of plant mitochondrial biogenesis may rather depend on the regulation of the level of nuclear-encoded proteins governing assembly of macromolecular complexes, as has been speculated for sucrose-starved Arabidopsis cell cultures [43]. Overall, temperature stress response seems to involve no more than ca. 5% mitochondrial proteins encoded by mtDNA.

Some proteins detected in our study were previously shown to vary under diverse abiotic stress conditions. PDH participates in regulation of carbon flux from glycolysis to TCA cycle. In the published data, upregulations of PDH subunits prevail; in pea (*Pisum sativum*) mitochondria, PDHβ proteolytic products accumulate [50,77,78]. In rice leaves, however, contrasting PDHα responses (similarly to HSP90, see below) were noted under diverse cold conditions [37]. It is known that also other components of PDH complex including dehydrolipoamide dehydrogenase, may be upregulated during heat stress [79]. In contrast, we showed extensive accumulation of PDHβ after cold recovery, but not after heat treatment. In rice, PDHβ was downregulated during hypoxia [80], however, subunit-α of this enzyme increased during heat treatment and decreased in abundance after stress cessation [81]. Our results suggest that despite the overall number of major cold-responsive mitochondrial proteins being lower than those regulated by heat, it seems that carbon transfer from glycolysis to TCA cycle is increased in cauliflower cold response. Nonetheless, stress can regulate plant energetic and metabolic demands, including ATP/ADP intracellular and intramitochondrial ratio and the need for carbon skeletons [82].

Mitochondrial NAD^+^-dependent MDH, which was increased in abundance after cold recovery in cauliflower mitochondria, in Arabidopsis was accumulated in response to different environmental stimuli including cold de-acclimation (but not cold acclimation) [32,33,83,84]. Arabidopsis MDH1 was suggested to belong to translational regulation targets [85]. The level of this enzyme (together with CS) was diversely modulated by various chilling conditions; generally, MDH abundance increased in cold-sensitive plant species [26,37,50,78,86,87]. Dumont et al. [88] investigating alterations in MDH abundance in diverse pea genotypes submitted to the combined cold and frost action and obtained contrasting results depending on the stress treatment and duration, similarly to the Yin et al. [89] and Cheng et al. [90] studies on MDH1 level in soybean (*Glycine max*) embryonic axes. Interestingly, CS responses depend on the severity of the temperature treatment (e.g., in the severe chilling the abundance of this enzyme declined), whereas under moderate treatment it increased [91]. During 2-day-long heat stress, MDH was also diversely downregulated in two *Agrostis* species depending on their thermotolerance [92]. Such a decrease in abundance was also reported for soybean MDH [89]. The significant up-regulation of cauliflower MDH only to cold recovery suggests that it may be the cold recovery marker [93]. However, heat recovery appeared detrimental for the level of this enzyme in cauliflower mitochondria [41]; cauliflower IDH and CS markedly declined after a 2-day-long heat recovery. Similar changes were reported for CS in heat adapted *Populus euphratica* [79]. In general, heat (which may lead to intramitochondrial oxidative damage) results in TCA enzymes, mitochondrial NADH pool and ATP synthesis impairments [94] and cold stress results in general stimulation of respiratory metabolism.

It appeared that cold stress causes an increase in the level of cauliflower HSPs; interestingly, in our study HSP70 and HSP90 increased more than in pea (*Pea sativum*) and rice leaves and peach (*Prunus persica*) barks [38,50,95]. A similar trend was observed in rice during salinity [96] and heat action in Arabidopsis [97]. However, mitochondrial HSP70 declined in abundance in stored or detached peach fruits submitted to prolonged cold [77,98]. Van Aken et al. [29] reported that Arabidopsis mitochondrial heat shock proteins responded only slightly to some forms of abiotic stress, for example HSP70 in the case of Cd treatment [83]. Another protein, HSP81-2, appeared to be cold-responsive in cauliflower mitochondria, contrary to the Arabidopsis ortholog, which was regulated by heat [31,97]. Notably, the regulation of HSP90 level in rice leaves depended on cold duration [38]. CPN10 remained unaffected after cold stress in pea [50], but in cauliflower mitochondria this protein accumulated very extensively under heat recovery. Also, mitochondrial sHsp22 was induced preferentially by heat (but not by cold) in soybean seedlings [25]. Together with FunCat data, all those findings suggest that the accumulation of some HSPs in cauliflower mitochondria may be specific for the preferential temperature stress conditions. Some HSPs can also diversely participate in various stress conditions, leading to distinct stress responses. It should also be noted, that the expression of two proteins (HSP70 and MDH1) regulated by low-temperature treatment, as well as additional proteins (mtEF-Tu, CS) responded to heat/heat recovery in our study and is known to be modulated by the specific glycine-rich protein (displaying RNA chaperone activity) under cold adaptation in Arabidopsis plants [99].

Cauliflower mtEF-Tu increased in abundance mainly after heat stress and did not last after heat recovery; overall, this may imply that the mitochondrial translation apparatus is impaired after heat cessation and rapid shift to control growth conditions of cauliflower, which was also observed for instance in chilled soybean embryo axes [89]. mtEF-Tu together with β-subunit of succinyl-CoA ligase increased in abundance in drought and partially in flood and MPP and ATP1 by salinity in Arabidopsis [32,84]. Curiously, β-subunit of succinyl-CoA ligase showed heat duration-dependent responses in soybean roots and rice leaves [25,37]. The major downregulation of succinyl-CoA ligase β-subunit in cauliflower mitochondria followed alterations of other TCA cycle components (IDH, CS) after heat stress [37,79], but not after heat recovery. Therefore, we can speculate that succinyl-CoA ligase may be preferentially accumulated in cauliflower during heat recovery in order to adjust the mitochondrial metabolism to control conditions.

ATP1 belongs to the proteins with level alterations dependent on the given species as well as stress intensity and duration [42]. In our study, ATP1 was declined in abundance after heat recovery. Similar trends were noted for pea, Arabidopsis, and *Zea mays* in a course of chilling, prolonged heat, CuCl or H_2_O_2_ treatment [39,50,100,101]. In contrast, cauliflower ATP1 abundance slightly increased in heat, similarly to the unassembled subunit *b* of ATP synthase [41]. We also found that heat caused a vast increase in abundance of ATP synthase d-subunit, contrary to its major downregulation reported by Gammulla et al. [37] and Tan et al. [39] for cold-stressed Arabidopsis cell cultures and heat-treated rice leaves, respectively. During oat (*Avena sativa*) seed storage, ATP1 level consistently declined as temperature increased from 35 to 50 °C, whereas subunit d of ATP synthase initially increased and then decreased in abundance under the same treatment; notably, subunits d and α were differentially accumulated at 10% and 16% moisture content, respectively [102]. Overall, those findings suggest that demand for ATP synthesis during heat treatment increases and the excess of de novo synthesized diverse ATP synthase subunits (e.g., mitochondrially encoded ATP1 or nuclear-encoded ATP7 proteins) is likely to be assembled into novel ATP synthase holocomplexes, labile in heat recovery [41].

Regarding the decrease in the level of MPPβ after heat recovery, we think that this may reflect the impairment of the import machinery, which may not be fully restored after stress recovery: according to our previous study [41], another subunit—MPPα appeared also to be down-regulated in heat recovery. Gammulla et al. [37] and Neilson et al. [38] noticed contrasting changes in the level of MPP subunits in rice leaves under low temperature and overall downregulations under heat. The level of MPP subunits underwent major changes in flood, indicating mitochondrial damage [84]. The influence of abiotic stresses on the efficiency of protein import into plant mitochondria was investigated, inter alia, by Taylor et al. [103] and Giegé et al. [43]. Taylor et al. [103] observed import inhibition of all tested pre-proteins into pea mitochondria during thermal stress. In turn, Giegé et al. [43] reported that the capacity for in vitro mitochondrial protein import is not affected after sucrose starvation in Arabidopsis cell cultures. Owing to our present and previous results [41], the pattern of protein import into cauliflower mitochondria under temperature stress should be investigated.

Another down-regulated cauliflower mitochondrial protein in heat was P5CDH, an enzyme involved in the proline degradation pathway of the Pro/P5C cycle [104]. Enzymes of this cycle, including P5C synthetase and proline dehydrogenase (ProDH) could be reciprocally expressed under stress. Moreover, ProDH closely associates with the OXPHOS system [42,105]; it was suggested that P5CDH prevents oxidative stress and electron run-off within the mitochondrial respiratory chain during Pro metabolism [106]. Free Pro accumulated in leaves of cold-treated cauliflower of wild type and mutant clones selected on hydroxyproline-containing medium, however, after salinity stress in mutated populations [107,108]. Interestingly, the level of *P5CDH* messengers significantly decreased in Arabidopsis plants expressing ectopically P5C synthetase 1 in response to heat stress. Pro accumulation impeded Arabidopsis seedlings growth in heat stress and may not serve as a protective osmolyte [109]. Therefore, it would be important to determine whether the decrease in abundance of P5CDH in cauliflower curds is associated with the increased Pro level after heat stress and heat recovery.

To extend our knowledge regarding cauliflower cold-responsive proteins, we carried out immunoblotting using antisera against dedicated proteins (Figure 3). We observed accumulation of cytosolic Hsp17.6C-CI after cold stress and recovery, indicating interaction of small HSPs with cauliflower mitochondrial membranes under prolonged cold treatment (as speculated by Rikhvanov et al. [54] for heat-stressed Arabidopsis cell cultures) and the importance of stress recovery phase in gaining stress resistance. Overall, HSPs are known to form oligomeric complexes with stress-affected proteins [110]. Important photorespiratory enzymes, GDC and SHMT, were decreased in abundance after cold recovery, similarly to *Agrostis scabra, A. stolonifera*, Arabidopsis, pea, *P. cathayana*, rice and wheat (*Triticum aestivum*) proteins in cold, heat and drought [37,38,50,92,111,112,113]. This observation is consistent with the reported declined levels of those enzymes in plant mitochondria under unfavourable conditions, leading to photorespiratory impairments [111]. However, under microspore development in rice plants submitted to cold, GDC-H was upregulated [114]. Interestingly, such up-regulation of GDC-H was also reported in pea leaves under frost and independently to cold tolerance and in the case of SHMT- in cold and salinity [37,88,115]. GDC-H slightly increases in abundance also in the early stages of low temperature action [116]. In accordance with that observation, as a part of protective mechanisms, significant accumulation of GDC-H transcripts in Arabidopsis leaves in response to short cold treatment was also reported [117].

We also determined variations in the level of some proteins engaged in maturation of cyt. *c*. Interestingly, major level downregulations of CcmF_N1_ and CcmF_N2_ proteins suggest that components of cyt. *c* maturation apparatus, including putative heme lyase components, may be sensitive to temperature stress. Generally, evidence for alterations of the level of those proteins in plant mitochondria during stress conditions are quite scarce. However, Naydenov et al. [118] found that *Ccm*F_N_ messengers responded during three-day-long cold exposure in maize embryos.

Regarding the level of other mitochondrial proteins during cold stress and cold recovery, VDAC-1 was downregulated in cauliflower mitochondria, which is generally in accordance with previously published results [37,77,94,119]. Interestingly, according to our previous study [41], we found the affected level of another VDAC isoform (VDAC-2) under heat recovery only. In addition, we detected some level of regulation of selected RC components, such as NAD9, COXII and cyt. *c*_1_. Despite Tan et al. [39] having found cyt. *c*_1_ abundance alterations (roughly followed by our data) among a number of Arabidopsis proteins in terms of their decline in cold, they did not identify COXII among them. However, those authors also reported the increased level of NAD9 protein in chilled Arabidopsis cell cultures. Longer cold acclimation led to the downregulation of this protein in wheat crowns, which is similar to our data [112], but 72 h-long cold stress resulted in NAD9 increase [37]. Selected CIV subunits [e.g., 6b-1 in chickpea (*Cicer arietinum*)] could also decline in abundance in cold, which suggests that overall respiratory activity decreased [120]. In most of the investigated plants, the level of COXII increased under low temperature and the overall changes in NAD9 abundance seem to be species-specific under temperature stress [5,121,122]. OXPHOS components are heat action sites in cauliflower [41]. It should be underlined that cold/cold recovery responses in cauliflower mitochondria also resulted in few protein upregulations, as was evident from 2D PAGE.

Finally, immunoblotting results extended the 2D PAGE data for cold-regulated proteins and the current study has also broadened knowledge on temperature stress responsive mitochondrial proteins, compared to our previous complexomic data [41]. Obtained data indicate for the variations of the same mitochondrial proteins in analysed stress conditions between cauliflower and other plant species. Few novel proteins, representing various pathways of mitochondrial metabolism, were discovered as responsive ones in thermal stress in cauliflower mitochondria. Therefore, one could speculate that numerous signalling pathways may be induced during action of cold or heat stress to alter the pattern of mitochondrial proteome. Various metabolic pathways (e.g., TCA cycle) may diversely participate in a particular stress response, which results in a plethora of various proteomic effects for cold, heat stress conditions and for stress recovery. In addition, the imbalance between proteomic and transcriptomic responses investigated in this study suggest that messengers accumulated at lowered levels have to be more efficiently used for translation, presumably by adaptive alterations in transcript/ribosome associations. Moreover, lack of coordination of expression profiles of diverse genes coding for subunits of the same complexes (CI, ATP synthase) in diverse temperature treatments points to the putative aberrations in the biogenesis of OXPHOS complexes also in cold and cold recovery and extends our previous data [41].

### 3.3. Cauliflower Leaf Respiratory Responses to Cold and Heat Stress

Temperature belongs to the critical factors controlling plant growth and development. Understanding both molecular, physiological as well as metabolic responses of crop and vegetable species to temperature stress in order to improve their tolerance and sustain high field yields is crucial [123]. However, the data regarding physiological functioning of *Brassica* species, including cauliflower, in cold and heat treatment [15,124] is still insufficient, contrary to some other environmental conditions, such as salinity or cadmium treatment [125,126,127,128,129].

Cauliflower is one of the most agriculturally important vegetable crops worldwide [107]. Notably, cauliflower and kale (*B. oleracea* var. *acephala*) belong to species better cold- and frost-adapted than Arabidopsis [107,130]. In our study, cold or heat stress was applied to cauliflower plants at the early stage of curd development, which enabled us to study the stress response of plants both at the molecular and physiological level. Previously, we used polarographic assays for investigating physiological properties and the activity of alternative pathway under temperature stress and recovery in isolated cauliflower mitochondria [41]. For physiological measurements in the current report, fully developed cauliflower leaves instead of curds were chosen; leaves, contrary to other plant organs, appeared more cold sensitive, which makes them most suitable for physiological assays [131]. We determined leaf respiration rate (by gas-exchange measurements on illuminated and darkened leaves), transpiration, stomatal opening, net CO_2_ assimilation rate as well as chlorophyll level and fluorescence parameters which appeared to be affected by the same treatments to various extents (Figure 5, Figure 6 and Figure 7), complementing our previous data.

In our study, the increase of R_n_ and R_d_ after heat stress and their subsequent decrease to the level of control variant during heat recovery suggest that adaptive forces of respiratory metabolism of cauliflower leaves to thermal treatment depends on stress duration. The increase of respiration after heat stress was also assayed in a number of plants [e.g., pepper (*Capsicum annuum*) leaves], which is a thermotolerant species with effective energy dissipation and ROS scavenging systems [132]. Due to the fact that cold stress acted for a longer period and appeared even more detrimental than heat, we did not observe R_n_ return to the level of control variant after cold recovery. This indicates for some irreversible effects in the cold, contrary to heat response (Figure 5). Such temperature recovery is expected to control energetic needs during acclimation, because of larger maintenance costs due to the increased activity of numerous enzymes [14].

The rate of respiration belongs to the first processes affected in plants also subjected to the low temperature treatments [27]. In the illuminated cauliflower leaves, R_d_ burst after cold recovery was evident; also for cold-acclimated Arabidopsis plants light respiration increased [14]. In some plants, however, the increase in respiration rate is visible at the early stage of cold treatment and it declines afterwards [42]. Overall, the importance of R_d_ in thermal adaptation is suggested. In addition, Talts et al. [14] showed that R_n_ of Arabidopsis leaves was more sensitive to cold stress than R_d_. We noticed a more evident decrease in R_d_ rather than in R_n_ after cold treatment (Figure 5). However, in various winter and spring wheat and rye (*Secale cereale*) cultivars, chilling also resulted in a small R_n_ increase [133]. Apart from the known various temperature treatments, species-specific respiratory responses are suggested between various plant species.

In our study, cold, heat and heat recovery resulted in a significant decrease of net photosynthesis rate, which was partially accompanied by decreased stomatal conductance [14,132,134,135]. Similarly, to cauliflower data, the post-cold plant acclimation resulted in recovery of photosynthesis [134]. Copolovici et al. [135] pointed out the relevance of various cold/heat treatments for different photosynthesis and stomatal conductance decrease in tomato (*Solanum lycopersicum*) leaves, which is also important in our case (Figure 6). Dahal et al. [133] showed that in some wheat and rye cultivars, cold resulted in a decrease of both net CO_2_ assimilation and as well as leaf transpiration and stomatal conductance. Leaf transpiration, stomatal conductance, chlorophyll content and photosynthesis also responded to cold in *P. cathayana* [136]. The decreased photosynthetic CO_2_ assimilation rate in cauliflower leaves also correlated with an apparent decrease in stomatal conductance and transpiration rate after cold stress; however, in heat stress, stomata were closed to an even greater extent (Figure 6). Similarly, heat stress affected photosynthetic parameters and decreased stomatal conductance in grapevine (*Vitis amurensis*) and tobacco leaves [137,138]; in cauliflower leaves after heat recovery, despite rapid stomatal opening, the net photosynthetic rate remained decreased (Figure 6).

Under temperature stress, chlorophyll level and fluorescence as well as PS performance could be affected to various extents [10,124,137,139]. In our case, the decrease of Fm and Fv was accompanied by lower amounts of chlorophyll in cauliflower leaves only after cold treatment, and chlorophyll fluorescence parameters were not restored only after heat recovery. The lower chlorophyll content in leaves of cold stressed plants may suggest some damage in photosynthetic apparatus because photosynthetic rate was decreased. Generally, heat stress may result in the decrease of Fv/Fm [132]. However, in our study, PSII performance was largely unaffected due to the overall stable Fv/Fm ratio in all stress conditions investigated (Figure 7). It is known that the heat damage of PSII, accompanied by a decrease of CO_2_ assimilation rate, occurs when severe stress conditions (exceeded 42 °C) were applied on illuminated leaves; however, this damage could be restored either in cases when a ‘point of no return’ is not exceeded or when exogenous Ca is applied for stomatal opening [138,140,141]. We conclude that despite our heat treatment conditions bordered with this threshold between mild and severe conditions, closed stomata in heat stress resulted in overall photosynthetic, but not respiratory decrease (Figure 5 and Figure 6) and overall less susceptibility of cauliflower to heat than cold treatment.

We also noticed an association between decreased photorespiration rate and GDC-H and SHMT levels in cold recovery (Figure 4 and Figure 5). Photorespiratory decline under temperature stress may result from GDC and SHMT downregulations in abundance and/or activity [50,92,111,117]. In our study, photorespiratory impairment was observed after heat treatment and heat recovery. Here, decreased photorespiration corresponded with decreased photosynthetic activity (due to over-reduction of the photosynthetic chain) and appeared irreparable after heat recovery. Also, in pepper leaves, heat treatment decreased both net photosynthetic as well as photorespiratory rate [132]. Interestingly, in cauliflower mitochondrial proteome, the increased level of MDH was associated with GDC-H and SHMT downregulation in cold stress and cold recovery. Mitochondrial MDH, which assists in metabolic flux through the TCA cycle, could operate in a reverse manner, by reducing oxaloacetate to malate, providing NAD^+^ for photorespiratory glycine decarboxylation [142]. Regarding our study, mitochondrial MDH did not respond to heat treatment and heat recovery. Despite distinct tissues (curds and leaves) being chosen for proteomic and physiological experiments, still we may speculate whether cauliflower NAD^+^-dependent MDH is engaged rather in the increase of the NADH pool inside mitochondria by acting within the TCA cycle, and not in NAD^+^ regeneration necessary for photorespiration in cold stress, because the level of MDH and GDC-H were regulated conversely. Further experimental attempts are necessary to elucidate this issue.

**Figure 8:**
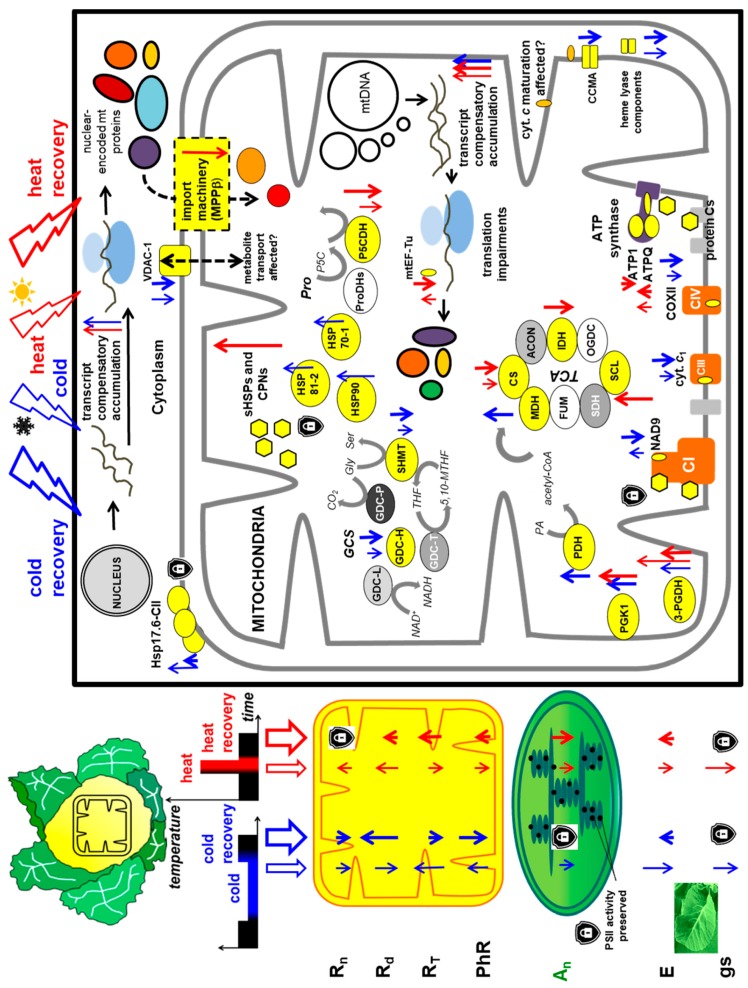
Proposed model of the impact of temperature stress and recovery on mitochondrial biogenesis in cauliflower curds. Investigated phenomena are depicted. Regulations in the cold and heat are shown by thin blue and red arrows; regulations in stress recovery by the respective thick blue and red arrows. Up-regulations and down-regulations are denoted by arrow heads raised up and down, respectively. Icons with a shield inside depict sustained physiological parameters or phenomena (**left panel**) or protective function of selected proteins in mitochondrial biogenesis (**right panel**). **Left panel:** stress dosage scheme and regulations in key physiological parameters. **Right panel:** Proteins and complex subunits regulated by abundance are yellow-marked. Some regulatory steps affected by stress conditions are highlighted by discontinuous lines and arrows. Important abbreviations: A, net photosynthetic rate; ATP1, ATPQ, ATP synthase subunits 1 and d; CCM, cytochrome *c* maturation; CPN, chaperonin; C(s), complex(es); COX, cytochrome *c* oxidase; CS, citrate synthase; cyt. *c*, cytochrome *c*; E, leaf transpiration; GCS, glycine cleavage system; GDC, glycine decarboxylase; gs, stomatal conductance; HSP(s), heat shock protein(s); IDH, isocitrate dehydrogenase; MDH, malate dehydrogenase; MPP, mitochondrial processing peptidase; mt, mitochondrial; NAD9, complex I subunit 9; P5CDH, 1-Δ-pyrroline-5-carboxylate dehydrogenase; PDH, pyruvate dehydrogenase; 3-PGDH, 3-phosphoglycerate dehydrogenase; PGK1, phosphoglycerate kinase isoform 1; PhR, photorespiration; ProDH(s), proline dehydrogenase(s); R_d_, light respiration; R_n_, dark respiration; R_T_, total light respiration; SCL, succinyl-CoA ligase; SHMT, serine hydroxymethyltransferase; TCA, tricarboxylic acid; VDAC, voltage-dependent anion channel. Further data in the text.

Figure 8 summarizes results of our study. In general, we suggest that distinct cold and heat stress responses act in various way not only on the cauliflower mitochondrial proteome, but also on investigated transcript alterations and physiological parameters related with respiration with limited association. As it was pointed out above, associations between photorespiration rate and the dedicated enzymes were clearly seen. Contrasting conditions of temperature stress and recovery may result in diversely affected pre-protein import to cauliflower mitochondria, impaired metabolite exchange and altered chaperoning activity together with TCA and OXPHOS functioning. In addition, transcript accumulation is proposed to compensate affected protein pool. Nevertheless, specificity of studied physiological and molecular responses to cold and heat stress between cauliflower and other plant species were easily observed and should be investigated in the future in more detail.

## 4. Materials and Methods

### 4.1. Plant Material, Growth Conditions and Stress Treatment

Cauliflower (*Brassica oleracea* var. *botrytis* subvar. *cauliflora* DC cv. ‘Diadom’) seeds were purchased from Bejo Zaden (Warmenhuizen, Holland). Cauliflower seedlings were grown in 0.09 dm^3^ pots filled with peat substrate for growing cruciferous vegetables (Kronen–Clasmann, Gryfice, Poland). Seedlings with 3–4 leaves were transferred to larger containers (5 dm^3^ in volume). Plants were grown for three months in cultivation chambers at a local breeding station (Poznan University of Life Sciences, Poland) at 23/19 °C (D/N) and 70% relative humidity under photon flux density 200 μmol·m^−2^·s^−1^ (16 h of light/8 h of dark). After three months of growth corresponding to the young inflorescence (10 cm in diameter) stage, plants were divided into a few sets for stress treatment and the parallel control variants (plants grown in conditions described above).

Two stress variants were tested in this study: the direct stress treatment-heat or cold and post-stress plant cultivation (stress recovery). For the application of cold stress, plants before the isolation of mitochondria were transferred for ten days to 8 °C. Heat treatment (40 °C) was applied to growing plants for 4 h before the isolation of mitochondria. After stopping the stress treatment, part of cauliflower plants were transferred to the standard growth conditions for 48 h for the stress recovery. Curds (5 mm topmost layer) were directly harvested either after stopping the stress treatment or after stress recovery.

### 4.2. Gas Exchange Measurements

All analyses were carried out on at least three fully developed leaves from three 3-month-old plants. Leaves were taken from each plant representing all experimental variants (control versus stress-treated or control versus stress recovered plants). At least three biological replicates were analyzed. All parameters (the rate of total CO_2_ assimilation [A_g_], A_n_, total respiration rate [R_T_], R_d_, R_n_, E, and g_s_) were measured using an LI-6400 XT infrared gas analyzer (LI-COR, Lincoln, NE, USA) and adjusted to the enclosed leaf area determined by an LI-300 leaf meter (LI-COR, Lincoln, NE, USA). Data were recorded after at least 2 h-long illumination. During the experiment, each of the analyzed leaves were placed into a 6-cm^2^ chamber of the analyzer. Results were recorded after initial leaf acclimation to the desired light and CO_2_ concentration, relative humidity and temperature. Gas-exchange parameters were recorded after leaf acclimated in the gas exchange chamber under the following conditions: PPFD of 400 μmol·m^−2^·s^−1^, 50% of the relative humidity (RH), 22 °C, 350 ppm of CO_2_. CO_2_ assimilation rate was also determined at two additional PPFD values (200 and 600 μmol·m^−2^·s^−1^).

R_d_ rate was determined according to Laisk [55]. For each leaf, CO_2_ assimilation rate representing a given R_T_ was recorded during decreasing intercellular CO_2_ concentration (C_i_) to 0 ppm at 22 °C and 50% RH, and for each of the three different PPFD values (200, 400 and 600 μmol·m^−2^·s^−1^). For each PPFD, the linear regression of CO_2_ assimilation (A) versus C_i_ was calculated (A/C_i_ curve) and the photorespiration rate (PhR for each PPFD value, denoted as PhR200, PhR400 and PhR600) was determined as the difference between R_T_ and R_d_ values (the last one expressed as a given CO_2_ evolution rate at the point of crossing of all A/C_i_ curves). The R_n_ rate was extrapolated from the A value during decreased PPFD to 0 μmol·m^−2^·s^−1^ from A/PPFD curve.

### 4.3. Chlorophyll Content and Fluorescence Measurements

Chlorophyll content was measured with a SPAD-502 chlorophyll meter (Konica Minolta, Wrocław, Poland) and expressed in relative units. Chlorophyll fluorescence was determined using a portable fluorometer (PAM-2000; Heinz Walz GmbH, Effeltrich, Germany) in a dark room with stable conditions. Before measurement, leaves were dark adapted for 30 min. Minimal fluorescence (Fo) was measured under 650 nm wavelength at a very low intensity (0.8 μmol·m^−2^·s^−1^). Fm was estimated after 1 s application of the saturating pulse of white light (3000 μmol·m^−2^·s^−1^). PSII photochemical efficiency was estimated from the Fv/Fm ratio, where Fv stands for the difference between Fm and Fo.

### 4.4. Preparation of Mitochondria

Mitochondria from 100 to 500 g of 5 mm-thick apical layer of cauliflower curds were isolated using a modified protocol of Boutry et al. [143], as described by Pawlowski et al. [23]. During isolation, the Complete Mini EDTA-free Protease Inhibitor Cocktail (Merck Poland, Warsaw, Poland) was added. Protein concentration was determined by the Bradford [144] method, using BSA as a calibrator.

### 4.5. Control Assays

Purity assays of isolated mitochondria (measurement of activities of mitochondrial cyt. *c* oxidase, peroxisomal catalase, plastid alkaline pyrophosphatase and cytoplasmic alcohol dehydrogenase) were conducted according to Pawlowski et al. [23]. Additionally, the purity of isolated mitochondria was verified by transmission electron microscopy (JEOL 1200EXII, Jeol, Peabody, MA, USA; [56]).

### 4.6. Preparation of Samples for Two-Dimensional Electrophoresis (2D SDS-PAGE)

Freshly isolated samples of cauliflower mitochondria were precipitated with trichloroacetic acid at −20 °C overnight [145,146]. After centrifugation for 5 min (16,000× *g*, 4 °C), pellets were washed once with 1 mL of acetone supplemented with 20 mM dithiothreitol (DTT) and re-centrifuged as described above. After vacuum drying, pellets were resuspended in the lysis buffer (7 M urea, 2 M thiourea, 0.5% (*w*/*v*) 3-((3-cholamidopropyl)dimethylammonio)-1-propanesulfonate (CHAPS), 1.5% (*w*/*v*) DTT, 0.5% (*v*/*v*) pharmalyte, pH 3–10) and protein concentration was determined either with the modified Bradford assay [147] or using a 2D Quant Kit (GE Healthcare, Warsaw, Poland).

### 4.7. 2D SDS-PAGE

All analyses were conducted at 15 °C; at least three biological replicates were analysed. Mitochondrial proteins (100 μg for silver nitrate staining or 500 μg for colloidal CBB) were first separated according to their charge on rehydrated Immobiline dry strips (24 cm, containing linear gradient of pH 3–10) with the rehydration buffer (8 M urea, 2% (*w*/*v*) CHAPS, 0.3% (*w*/*v*) DTT, 2% (*v*/*v*) pharmalyte, pH 3 to10) on an IPGphor apparatus (GE Healthcare, Uppsala, Sweden). Conditions for isoelectrofocusing (IEF) were as follows: 1 h at 500 V (step), 1 h at 1000 V (gradient), 3 h at 8000 V (gradient) and, finally, 5.5 h at 8000 V (step). The strips were either stored at −80 °C or they were directly treated for 10 min with solution A (6 M urea, 50 mM Tris-HCl, pH 6.8, 30% (*v*/*v*) glycerol, 2% (*w*/*v*) SDS, 0.25% [*w*/*v*] DTT) and for the same time with solution B (solution A supplemented with 4.5% (*w*/*v*) iodoacetamide without DTT) and subjected for the second dimension run (SDS-PAGE).

For SDS-PAGE precast Ettan DALT 12.5% (*w*/*v*) polyacrylamide gels (GE Healthcare) and an Ettan Dalt Six electrophoretical chamber (for six gels) were used. Conditions for the run were as follows: 45 min at 80 V and 15 h at 120 V. After electrophoresis, proteins on gel triplicates were either silver stained [148] for protein variation analysis or stained with colloidal CBB, according to Neuhoff et al. [149] for MS analyses. 2D gels were scanned, analysed using 2D Image Master 7 Platinum software (GE Healthcare) and the normalized quantitative volume of protein spots was determined.

### 4.8. Statistical Analysis of 2D Protein Pattern Variations

Protein spots showing variations in abundance were submitted to ANOVA to select spots for which stress treatment of post-stress plant cultivation had a significant effect (*p* < 0.05) on their volume. Additionally, the most variable proteins were also checked using Tukey’s HSD test (JMP Software v8, SAS Institute, Cary, NC, USA). These variable proteins were further identified by MS.

### 4.9. Protein Identification by MS

For MS analysis, gel spots were subjected to a standard ‘*in-gel*’ digestion procedure during which proteins were reduced with 100 mM (*w*/*v*) DTT (for 30 min at 56 °C), alkylated with iodoacetamide (45 min at room temperature in the dark) and digested overnight with trypsin (sequencing Grade Modified Trypsin—Promega V5111). Resulting peptides were eluted from the gel with 0.1% (*v*/*v*) trifluoroacetic acid, 2% (*v*/*v*) acetonitrile.

Peptide mixtures were separated by liquid chromatography prior to molecular mass measurements (LC coupled to a linear ion trap-Fourier transform ion cyclotron resonance (LTQ-FTICR) mass spectrometer) on Orbitrap Velos mass spectrometer (Thermo Electron Corporation, San Jose, CA, USA) at the Mass Spectrometry Laboratory (Institute of Biochemistry and Biophysics, Polish Academy of Sciences, Warsaw, Poland). Peptide mixture was applied to an RP-18 precolumn (nanoACQUITY Symmetry^®^ C18—Waters 186003514, Waters, Warsaw, Poland) using water containing 0.1% (*v*/*v*) trifluoroacetic acid as mobile phase and then transferred to a nano-HPLC RP-18 column (nanoACQUITY BEH C18—Waters 186003545) using an acetonitrile gradient (0 to 60% (*v*/*v*) acetonitrile for 120 min) in the presence of 0.05% (*v*/*v*) formic acid with a flow rate of 150 nL·min^−1^. The column outlet was directly coupled to the ion source of the spectrometer working in the regime of data dependent MS to MS/MS switch. A blank run ensuring lack of cross contamination from previous samples preceded each analysis.

Acquired raw data were processed by Mascot Distiller followed by Mascot search (Matrix Science, London, UK, 8-processor on-site license) against NCBInr (version 20100203) with taxonomy restricted to *Viridiplantae*. Search parameters for precursor and product ions mass tolerance were 40 ppm and 0.8 Da, respectively, with allowance made for one missed trypsin cleavage, and the following fixed modifications: cysteine carbamidomethylation and allowed variable modifications: lysine carbamidomethylation and methionine oxidation, serine, threonine and tyrosine phosphorylation as well as deamidations, methylations, formylations and ethylations. Peptides with Mascot Score exceeding the threshold value corresponding to <5% False Positive Rate, calculated by Mascot procedure, were considered to be positively identified. Phosphorylation sites were predicted by PhosPhAt v4.0 (Available online: http://phosphat.uni-hohenheim.de/; [150]), NetPhos v2.0, available online: (http://www.cbs.dtu.dk/services/NetPhos-2.0/; [151]) and MUsite v1.0 (Available online: www.musite.net; [152]). Methylation sites were predicted by PMes (Available online: http://bioinfo.ncu.edu.cn/inquiries_PMeS.aspx; [153]). The data were compared with Arabidopsis data at PPDB (Available online: http://ppdb.tc.cornell.edu/dbsearch/searchmod.aspx). PPDB experimental sources concerned Zybailov et al. [154] as well as Kim et al. [155] data. Additional modified residues were predicted by FindMod (Available online: https://web.expasy.org/findmod/; [156]).

### 4.10. SDS-PAGE and Immunoblotting

Aliquots containing 20 μg of mitochondrial proteins were separated by 12% (*w*/*v*) SDS-PAGE [157]. For immunoassays, proteins were electroblotted from 1D (SDS-PAGE) or 2D (IEF/SDS-PAGE) gels onto polyvinylidene difluoride Immobilon-P membranes (Merck, Warsaw, Poland), using a Sedryt semidry blotting apparatus (Kucharczyk, Warsaw, Poland). Membranes were CBB-stained to ensure that equal amounts of proteins were transferred. After destaining and subsequent blocking of the membrane, they were incubated overnight with antibodies. Indicated antibodies (Table S4) were purchased from Agrisera (Vännäs, Sweden). Antibodies against cyt. *c*_1_ and ATP1 were kindly donated by Prof. Gottfried Schatz (University of Basel). Hsp17.6 antisera were a generous gift from Prof. Elisabeth Vierling (University of Massachusetts, Amherst, MA, USA). Antibodies against NAD9 and CCMA were produced by [158] and [159], respectively. CcmF_N1_ and CcmF_N2_ antisera were generated by [160]. Bound sera were detected using an anti-rabbit immunoglobulin G horseradish peroxidase or alkaline phosphatase conjugate diluted to 1/10,000 (BioRad Polska, Warsaw, Poland) and visualized with enhanced chemiluminescent reagents (GE Healthcare, Warsaw, Poland) or with Lumi-Phos WB Chemiluminescent Substrate (Life Technologies Poland, Warsaw, Poland). Immunoblotting images in triplicates were analyzed by Multi Gauge (v2.2, Fujifilm, Tokio, Japan) software and the representative pattern was presented. Band intensities were calibrated to the protein loading in the linear relationship (the control denoted as 1.00); the other bands were calculated relative to this value.

### 4.11. RNA Isolation and RT-semiqPCR

Total RNA from cauliflower curds was extracted using Trizol reagent or an EZ-10 Spin Column Plant RNA Mini-Preps Kit (BioBasic, Markham, ON, Canada) according to the manufacturer’s protocol. Genomic DNA contaminants were removed by RQ1 DNase I free of RNase (Promega Poland, Warsaw, Poland). cDNA was synthesized using 1 μg of RNA, 0.2 μg of random hexamers mixture from HexaLabel DNA Labeling Kit (Thermo Scientific, Gdańsk, Poland) and 200 units of M-MLV reverse transcriptase (Promega Poland, Warsaw, Poland) in a 20 μL total volume for 1 h at 37 °C. After first strand synthesis, the reaction mixture was diluted with 10 mM Tris-HCl, pH 8.0 three or six times; after normalization, aliquots of 1–2 μL were subjected to RT-semiquantitative multiplex PCR (RT-semiqPCR) in a 15 μL total volume.

RT-semiqPCR was performed in an Applied Biosystems 2720 thermal cycler (Applied Biosystems Poland, Warsaw, Poland) with the following profile: 3 min at 95 °C followed by 25–26 cycles depending on amplicon of 20 s at 95 °C, 30 s at 55 °C (except 58 °C and 50 °C for *coxII* and *CPN10*, respectively) and 30 s at 72 °C, and with a final incubation for 5 min at 72 °C. PCR products were separated on a 1.5% agarose gel and stained with ethidium bromide. The gels were documented using a GBOX XL1.4 (TK Biotech, Warsaw, Poland) imaging system and quantified with Multi Gauge (v.2.2, Fujifilm, Tokio, Japan). For RT-PCR assays, two biological and at least three technical replicates were included.

Cauliflower cDNA fragments for selected mitochondrial proteins were amplified using specific primers (Table S5); a 239-bp fragment of cauliflower actin1 (ACT1) cDNA was used as an internal standard. The amplicons were directly sequenced bi-directionally (Big Dye Terminator v.3.1 Cycle Sequencing kit, Applied Biosystems Poland, Warsaw, Poland) on an ABI Prism 31–30 XL system (Applied Biosystems Poland, Warsaw, Poland) for sequence identity verification.

## 5. Conclusions

Our approach comprises general data about variations regarding cold and heat stress responses in the mitochondrial proteome of cauliflower and in physiological parameters, related particularly to plant respiration. It appeared that the set of cauliflower mitochondrial proteins responded to temperature stress conditions as well as to the stress recovery varied from the previously described ones. These results significantly extend the deposited data also by means of investigated quantitative alterations. However, investigated proteomic, transcriptomic and respiratory physiological responses related to the functioning of cauliflower mitochondria in stress were largely not associated. For instance, the rates of respiration in illuminated leaves together with leaf transpiration and photorespiration were significantly affected by cold and/or cold recovery, despite more proteins of various functional classes being involved in heat/heat recovery. Studied transcripts and protein alterations in temperature stress and recovery involve contrasting responses. Interestingly, the expression patterns of genes coding for various CI and ATP synthase subunits also differ. According to our previous data [41], this may suggest perturbations in the biogenesis of OXPHOS complexes. Owing to the scarce representation of cold- and heat-affected proteins encoded in the mitochondrial genome during mitochondrial response to temperature stress and recovery, modulation of cauliflower mitochondrial biogenesis under the investigated stimuli may depend rather on the massive regulation of nuclear-encoded proteins.

We would like to emphasize that heat-regulated proteins were distinct (with minor exceptions) from the ones regulated by cold/cold recovery. Overall, we (1) noticed the impaired photorespiration rate which was followed by alterations in photorespiratory enzymes after cold recovery; (2) suggested possible metabolic impairments in various TCA components and Pro catabolism (downregulations of *PRODH* and *P5CDH* transcripts in cold and heat recovery were also notable), and in protein import apparatus; (3) observed elevated demand for ATP synthesis after heat/heat recovery (e.g., ATP1 and ATPQ level); (4) noticed evident downregulation of some RC subunits (e.g., ATP1, NAD9, COXII) and the sensitivity of *c*-type cytochrome biogenesis apparatus to cold stress and cold recovery; and (5) compared selected proteomic and transcriptomic responses providing additional data on their participation in temperature stress and recovery. Our data show that selected regulations cannot be fully restored after temperature recovery. All these results imply the necessity (1) to go deeper in the quantitative analysis of protein posttranslational modifications and (2) to study further tissue-specific proteomic and physiological alterations.

## Figures and Tables

**Table 1:** List of cauliflower mitochondrial proteins the level of which varied during stress treatments.

Spot No. ^a^	Mean % Volume ^b^			Assignment; Species; FunCat ^c^	Protein Record Version	UniProt Accession No.	AGI Identifier	Nominal		Observed		Mascot Score; emPAI ^d^	Coverage (%)	Uniq. Peps; Tot. Peps ^e^
	K	C; H	CA; HA					M	pI	M	pI			
1	0.53 ± 0.02	**1.06 ± 0.08 (+2.00)**	0.68 ± 0.19 [+1.28]	Mitochondrial heat shock protein 70-1; Arabidopsis; **PrF**	CAB37531.1	Q9SZJ3	At4g37910	71.4	5.31	79	5.35	4732; 3.61	33	53; 129
2	0.11 ± 0.01	0.21 ± 0.06 [+1.91]	**0.22 ± 0.03 (+2.00)**	Pyruvate dehydrogenase E1 beta subunit; Arabidopsis; **CM**	NP_199898.1	Q38799	At5g50850	39.4	5.67	39	5.16	5484; 2.94	29	73; 146
3	0.18 ± 0.01	**0.40 ± 0.09 (+2.22)**	0.21 ± 0.01 (+1.17)	3-phosphoglycerate dehydrogenase-like protein; Arabidopsis; **AM**	NP_195146.1	O49485	At4g34200	63.6	6.16	72	5.43	7707; 1.48	31	112; 266
4	0.11 ± 0.01	0.15 ± 0.01 (+1.36)	**0.21 ± 0.03 (+1.91)**	Phosphoglycerate kinase 1; Arabidopsis; **CM**	NP_187884.1	Q9LD57	At3g12780	50.1	5.91	43	5.20	630; 1.44	28	9; 26
5	0.15 ± 0.01	0.19 ± 0.04 (+1.27)	**0.29 ± 0.03 (+1.93)**	Malate dehydrogenase (NAD), mitochondrial; Arabidopsis: **CM**	NP_564625.1	Q9ZP06	At1g53240	36.0	8.54	37	5.58	409; 0.98	22	10; 18
6	0.19 ± 0.02	**0.37 ± 0.05 (+1.95)**	0.16 ± 0.03 (−1.18)	Heat shock protein 81-2 (HSP 90 related); Arabidopsis; **PrF**	NP_200414.1	P55737	At5g56030	80.2	4.95	84	5.20	277; 0.43	14	0; 12
7	0.16 ± 0.05	**0.39 ± 0.09 (+2.44)**	0.13 ± 0.07 (−1.23)	Heat shock protein 90; Arabidopsis; **PrF**	BAF00175.1	Q0WRS4	At3g07770	90.8	5.26	89	5.06	1996; 1.11	19	15; 75
8	0.17 ± 0.02	**0.64 ± 0.19 (+3.76)**	**0.48 ± 0.07 (+2.82)**	3-phosphoglycerate dehydrogenase-like protein; Arabidopsis; **AM**	NP_195146.1	O49485	At4g34200	63.6	6.16	73	5.56	6778; 1.74	30	88; 237
9	0.25 ± 0.03	0.72 ± 0.31 (+2.88)	**0.72 ± 0.16 (+2.88)**	3-phosphoglycerate dehydrogenase-like protein; Arabidopsis; **AM**	NP_195146.1	O49485	At4g34200	63.6	6.16	74	5.49	7997; 1.36	27	111; 301
10	0.05 ± 0.03	0.06 ± 0.01 (+1.20)	**0.19 ± 0.04 (+3.80)**	Phosphoglycerate kinase 1; Arabidopsis; **CM**	NP_187884.1	Q9LD57	At3g12780	50.2	5.91	81	5.50	1376; 2.57	42	22; 53
11	0.14 ± 0.08	0.12 ± 0.11 (−1.17)	**1.29 ± 0.25 (+9.21)**	Putative succinyl-CoA ligase (GDP-forming) beta subunit, mitochondrial; Arabidopsis; **CM**	NP_179632.1	O82662	At2g20420	45.6	6.30	41	5.15	265; 0.88	17	11; 11
12	0.56 ± 0.15	0.32 ± 0.17 (−1.75)	**0.11 ± 0.02 (−5.09)**	Putative mitochondrial processing peptidase; Arabidopsis; **PrF**	BAE98412.1	Q42290	At3g02090	51.5	5.71	80	5.79	833; 1.38	26	17; 42
13	0.36 ± 0.14	0.47 ± 0.09 (+1.30)	**0.19 ± 0.04 (−1.89)**	ATPase subunit 1; *Brassica napus*; **RC**	YP_717155.1	Q6YSN4	AtMg01190	55.4	6.01	58	6.07	3195; 1.12	23	76; 168
14	0.35 ± 0.14	0.22 ± 0.04 (−1.59)	**0.08 ± 0.03 (−4.37)**	Δ-1-pyrroline-5-carboxylate dehydrogenase precursor; Arabidopsis; **AM**	AAK73756.1	Q8VZC3	At5g62530	62.2	6.26	70	6.24	784; 0.67	18	3; 41
15	0.54 ± 0.06	**0.33 ± 0.06 (−1.64)**	**0.09 ± 0.01 (−6.00)**	Δ-1-pyrroline-5-carboxylate dehydrogenase precursor; Arabidopsis; **AM**	AAK73756.1	Q8VZC3	At5g62530	62.2	6.26	69	6.33	1622; 0.95	21	0; 78
16	0.60 ± 0.14	0.77 ± 0.11 (+1.28)	**0.31 ± 0.05 (−1.93)**	Mitochondrial elongation factor Tu; Arabidopsis; **PrS**	CAA61511.1	Q9ZT91	At4g02930	51.6	5.53	42	6.00	3123; 2.45	37	24; 100
17	0.31 ± 0.08	**0.42 ± 0.07 (+1.35)**	0.18 ± 0.04 (−1.72)	Mitochondrial elongation factor Tu; Arabidopsis; **PrS**	CAA61511.1	Q9ZT91	At4g02930	51.6	5.53	42	6.19	2502; 2.05	33	35; 77
18	0.58 ± 0.15	0.52 ± 0.07 (−1.11)	**0.14 ± 0.02 (−4.14)**	Isocitrate dehydrogenase-like protein; Arabidopsis; **CM**	CAB87626.1	Q9LYK1	At5g14590	52.3	7.11	45	6.37	697; 0.96	23	14; 23
19	0.26 ± 0.06	**0.17 ± 0.03 (−1.53)**	**0.11 ± 0.01 (−2.36)**	Citrate synthase (SI); Arabidopsis; **CM**	NP_850415.1	P20115	At2g44350	53.1	6.41	48	6.57	999; 0.62	18	34; 40
20	0.40 ± 0.09	**0.23 ± 0.07 (−1.74)**	**0.15 ± 0.01 (−2.67)**	Citrate synthase (SI); Arabidopsis: **CM**	NP_850415.1	P20115	At2g44350	53.1	6.41	47	6.83	2126; 0.83	21	51; 84
21	0.08 ± 0.06	0.16 ± 0.08 (+2.00)	**0.61 ± 0.08 (+7.62)**	10 kDa chaperonin; Arabidopsis; **PrF**	NP_563961.1	P34893	At1g14980	10.8	6.74	19	9.38	107; 1.30	31	8; 8
22	LA^f^	**0.80 ± 0.20**	**0.53 ± 0.01**	ATP synthase, d chain, mitochondrial; Arabidopsis; **RC**	NP_190798.1	Q9FT52	At3g52300	19.6	5.09	20	4.59	979; 1.60	25	18; 39

For each spot, values for nominal (computed) and observed isoelectric point (**pI**) and molecular mass (**M**, in kDa) and the mean normalized volume at each of analysed stress and control variants were indicated; ^a^ Spot number as index in the reference gel; ^b^ Mean value with the standard deviation of three spot volumes at each analysed stage: control (**K**), cold stress (**C**; **spots 1–7**), heat stress (**H**; **spots 8–22**), cold recovery (**CA**; **spots no. 1–7**), heat recovery (**HA**; **spots 8–22**). In parentheses, the fold change of mean value for stress variants, compared to control. Up-regulations are indicated by + and down-regulations by −. Significant regulations (according to HSD test) are **bolded** and underlined; the ones for cold, cold recovery, heat and heat recovery are shown in blue, green, red and brown highlights, respectively; ^c^ In **bold**: functional categorisation (FunCat) using data from FunCat scheme (Available online: http://ibis.helmholtz-muenchen.de/funcatDB/). Two-letter legend to all categories: **CM**—carbohydrate metabolism, **PrF**—protein fate, **AM**—amino-acid metabolism, **RC**—respiration (respiratory chain components), **PrS**—protein synthesis; ^d^ Exponentially modified protein abundance index; ^e^ Uniq. peps; tot. peps-number of unique peptides; number of total peptides for each protein spot; ^f^ LA—low-abundant protein (with the % volume of circa 0.01); in this case the calculation of the fold change was not applicable. Further experimental details in Materials and Methods.

## Supplementary Materials

Supplementary materials can be found at http://www.mdpi.com/1422-0067/19/3/877/s1.

## Abbreviations

A_n_	net CO_2_ assimilation rate
A_g_	total CO_2_ assimilation rate
ANOVA	analysis of variance
AOX	alternative oxidase
ATP1	ATP synthase subunit α
BSA	bovine serum albumin
CBB	Coomassie Brilliant Blue
Ccm/CCM	cytochrome *c* maturation
CAPS	3-[(3-cholamidopropyl)dimethylammonio]-1-propanesulfonate
C_i_	intercellular CO_2_ concentration
COX	cytochrome *c* oxidase
CPN	chaperonin
CS	citrate synthase
2D PAGE	two-dimensional gel electrophoresis
DTT	dithiothreitol
E	transpiration
EDTA	ethylenediaminetetraacetic acid
EGTA	ethylene glycol-bis(β-aminoethyl ether)-*N*,*N*,*N*′,*N*′-tetraacetic acid
EF	elongation factor
Fm	maximal fluorescence
Fo	minimal fluorescence
FunCat	functional categorization
Fv	variable fluorescence
GDC	glycine decarboxylase
g_s_	stomatal conductance
HSD	honest significant difference
HSP	heat shock protein
IDH	isocitrate dehydrogenase
IEF	isoelectrofocusing
iTRAQ	isobaric tags for the absolute quantification
LC-MS/MS	liquid chromatography-tandem mass spectrometry
MDH	malate dehydrogenase
MIPS	Munich Information Center for Protein Sequences
MPP	mitochondrial processing peptidase
NAD	complex I subunit (mitochondrially-encoded)
NCBI	National Center for Biotechnology Information
OXPHOS	oxidative phosphorylation
P5CDH	Δ-1-pyrroline-5-carboxylate dehydrogenase
PDH	pyruvate dehydrogenase
PGDH	3-phosphoglycerate dehydrogenase
PGK	phosphoglycerate kinase
PhR	photorespiration rate
PPFD	photosynthetic photon flux density
PRODH	proline dehydrogenase
PS	photosystem
PTM	posttranslational protein modification
RC	respiratory chain
R_d_	respiration in the light (day respiration) rate
RH	relative humidity
R_n_	respiration in the dark (night respiration) rate
ROS	reactive oxygen species
R_T_	total respiration rate
SCL	succinyl-CoA ligase
SHMT	serine hydroxy-methyl aminotransferase
TCA	tricarboxylic acid
VDAC	voltage-dependent anion channel
