# Supplementary material for: Cold and Heat Stress Diversely Alter Both Cauliflower Respiration and Distinct Mitochondrial Proteins Including OXPHOS Components and Matrix Enzymes
Source: Int J Mol Sci. 2018 Mar 16;19(3):877. doi: 10.3390/ijms19030877 (PMC5877738; doi:10.3390/ijms19030877)
Supplement: Supplementary file 1 [file ijms-19-00877-s001.zip › Figure S3.pptx]

## Slide 1
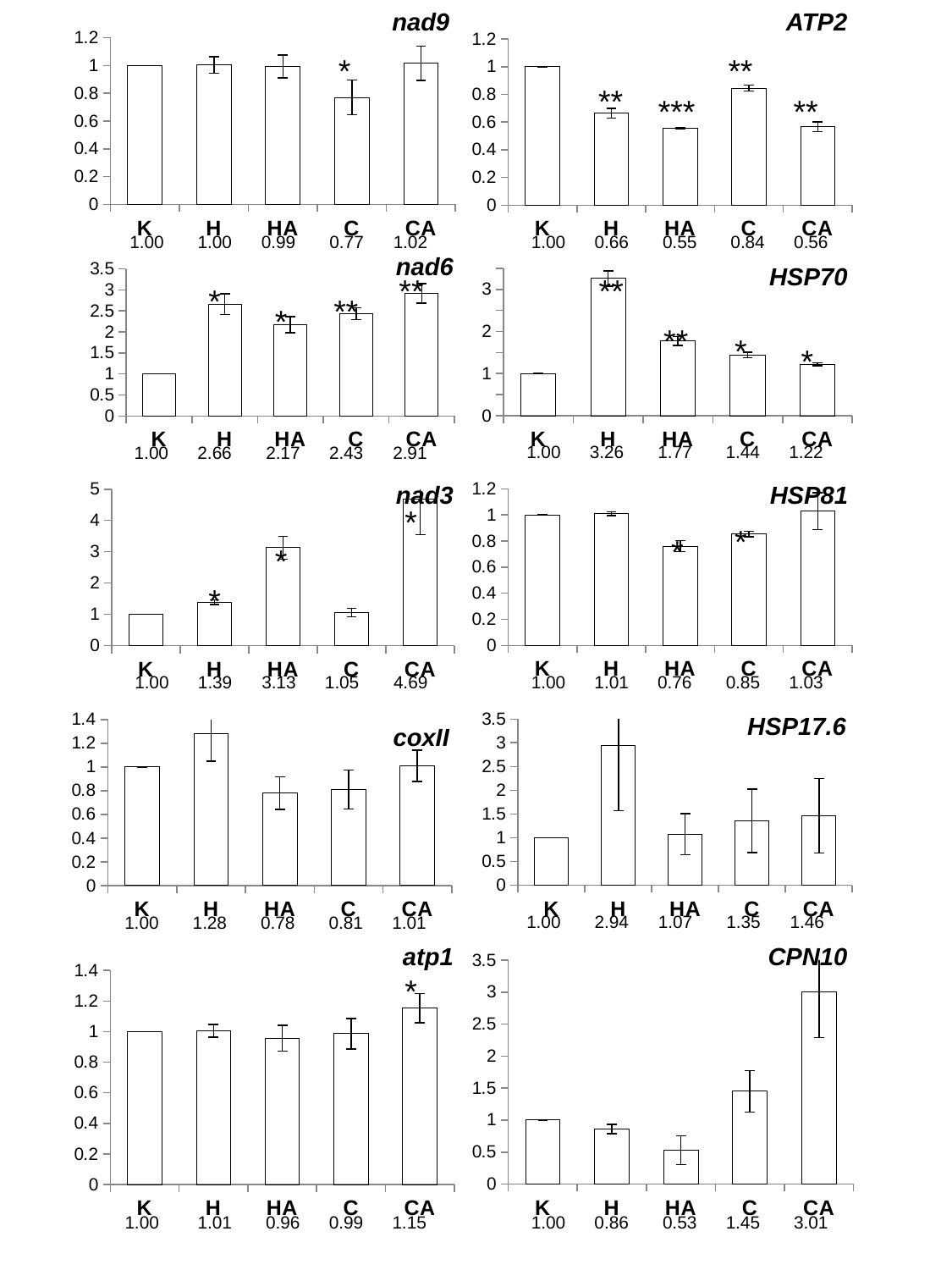

nad9
ATP2
### Chart
| Category | |
|---|---|
| K | 1.0 |
| H | 1.0033333333333332 |
| HA | 0.9933333333333333 |
| C | 0.77 |
| CA | 1.0166666666666666 |
### Chart
| Category | |
|---|---|
| K | 1.0 |
| H | 0.665 |
| HA | 0.555 |
| C | 0.845 |
| CA | 0.565 |*
**
**
***
**
 1.00 0.66 0.55 0.84 0.56
 1.00 1.00	 0.99 0.77	 1.02
nad6
HSP70
### Chart
| Category | |
|---|---|
| K | 1.0 |
| H | 2.665 |
| HA | 2.175 |
| C | 2.4299999999999997 |
| CA | 2.915 |
### Chart
| Category | |
|---|---|
| K | 1.0 |
| H | 3.26 |
| HA | 1.775 |
| C | 1.4449999999999998 |
| CA | 1.225 |**
**
*
**
*
**
*
*
1.00 3.26 1.77 1.44 1.22
 1.00 2.66	 2.17 2.43	 2.91
HSP81
nad3
### Chart
| Category | |
|---|---|
| K | 1.0 |
| H | 1.01 |
| HA | 0.76 |
| C | 0.855 |
| CA | 1.03 |
### Chart
| Category | |
|---|---|
| K | 1.0 |
| H | 1.3900000000000001 |
| HA | 3.13 |
| C | 1.055 |
| CA | 4.6899999999999995 |*
*
*
*
*
 1.00 1.01 0.76 0.85 1.03
1.00 1.39	3.13 1.05	 4.69
### Chart
| Category | |
|---|---|
| K | 1.0 |
| H | 2.945 |
| HA | 1.075 |
| C | 1.355 |
| CA | 1.465 |HSP17.6
### Chart
| Category | |
|---|---|
| K | 1.0 |
| H | 1.2766666666666666 |
| HA | 0.7800000000000001 |
| C | 0.81 |
| CA | 1.01 |coxII
1.00 2.94	 1.07 1.35	 1.46
 1.00 1.28 0.78 0.81 1.01
CPN10
atp1
### Chart
| Category | |
|---|---|
| K | 1.0 |
| H | 0.8600000000000001 |
| HA | 0.53 |
| C | 1.45 |
| CA | 3.01 |
### Chart
| Category | |
|---|---|
| K | 1.0 |
| H | 1.0066666666666666 |
| HA | 0.9566666666666667 |
| C | 0.9866666666666668 |
| CA | 1.1533333333333333 |*
 1.00 0.86 0.53 1.45 3.01
1.00 1.01 0.96 0.99 1.15

## Slide 2
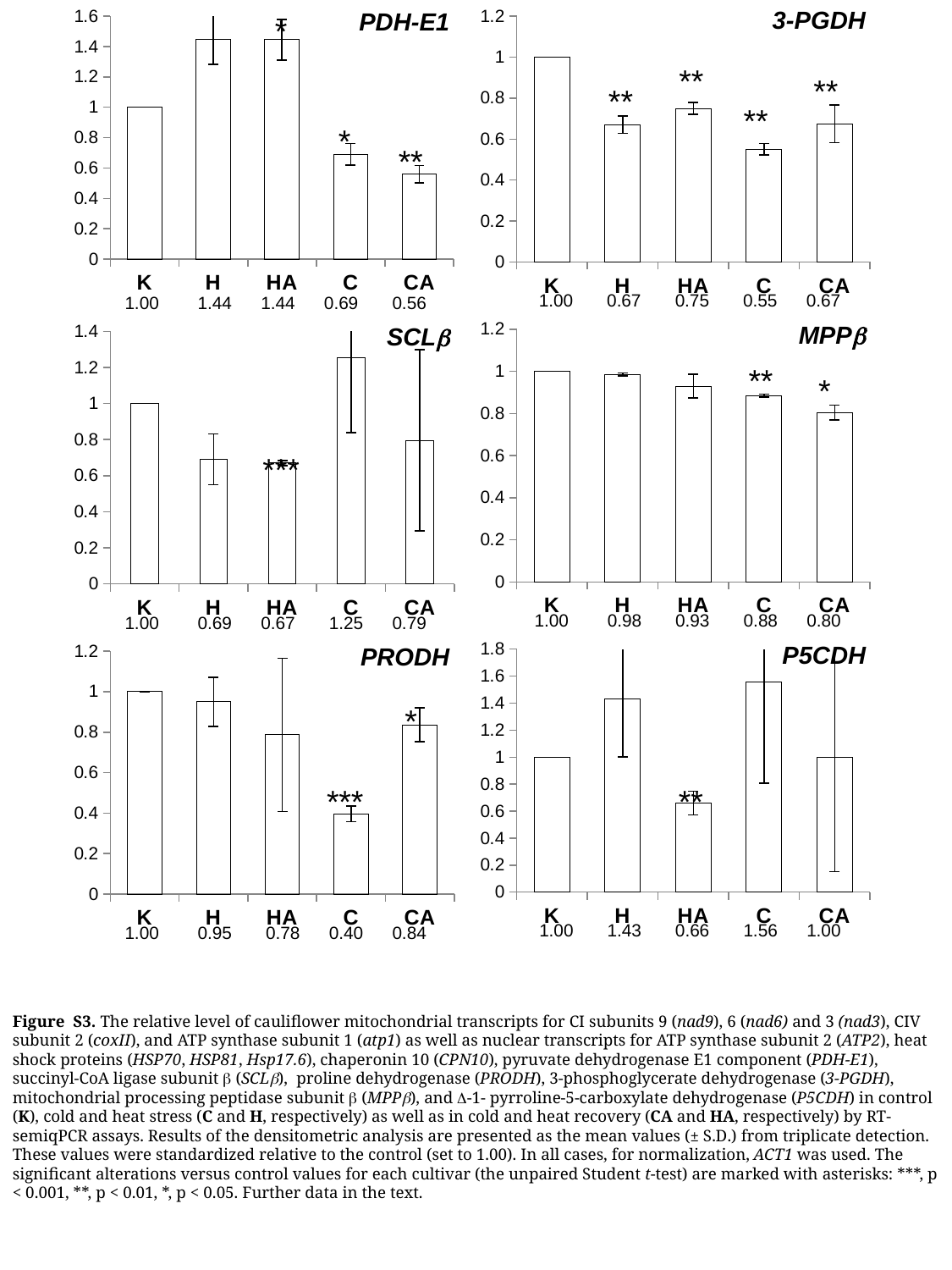

3-PGDH
### Chart
| Category | |
|---|---|
| K | 1.0 |
| H | 1.445 |
| HA | 1.445 |
| C | 0.69 |
| CA | 0.56 |PDH-E1
### Chart
| Category | |
|---|---|
| K | 1.0 |
| H | 0.6699999999999999 |
| HA | 0.75 |
| C | 0.55 |
| CA | 0.675 |*
**
**
**
**
*
**
1.00 0.67 0.75 0.55 0.67
1.00 1.44 1.44 0.69 0.56
### Chart
| Category | |
|---|---|
| K | 1.0 |
| H | 0.985 |
| HA | 0.9299999999999999 |
| C | 0.885 |
| CA | 0.8049999999999999 |MPPb
### Chart
| Category | |
|---|---|
| K | 1.0 |
| H | 0.69 |
| HA | 0.67 |
| C | 1.255 |
| CA | 0.7949999999999999 |SCLb
**
*
***
1.00 0.98 0.93 0.88 0.80
1.00 0.69 0.67 1.25 0.79
### Chart
| Category | |
|---|---|
| K | 1.0 |
| H | 1.4333333333333333 |
| HA | 0.66 |
| C | 1.5566666666666666 |
| CA | 1.0 |P5CDH
### Chart
| Category | |
|---|---|
| K | 1.0 |
| H | 0.9500000000000001 |
| HA | 0.7866666666666666 |
| C | 0.39666666666666667 |
| CA | 0.8366666666666666 |PRODH
*
***
**
1.00 1.43 0.66 1.56 1.00
1.00 0.95 0.78 0.40 0.84
Figure S3. The relative level of cauliflower mitochondrial transcripts for CI subunits 9 (nad9), 6 (nad6) and 3 (nad3), CIV subunit 2 (coxII), and ATP synthase subunit 1 (atp1) as well as nuclear transcripts for ATP synthase subunit 2 (ATP2), heat shock proteins (HSP70, HSP81, Hsp17.6), chaperonin 10 (CPN10), pyruvate dehydrogenase E1 component (PDH-E1), succinyl-CoA ligase subunit b (SCLb), proline dehydrogenase (PRODH), 3-phosphoglycerate dehydrogenase (3-PGDH), mitochondrial processing peptidase subunit b (MPPb), and Δ-1- pyrroline-5-carboxylate dehydrogenase (P5CDH) in control (K), cold and heat stress (C and H, respectively) as well as in cold and heat recovery (CA and HA, respectively) by RT-semiqPCR assays. Results of the densitometric analysis are presented as the mean values (± S.D.) from triplicate detection. These values were standardized relative to the control (set to 1.00). In all cases, for normalization, ACT1 was used. The significant alterations versus control values for each cultivar (the unpaired Student t-test) are marked with asterisks: ***, p < 0.001, **, p < 0.01, *, p < 0.05. Further data in the text.
