# Supplementary material for: Cold and Heat Stress Diversely Alter Both Cauliflower Respiration and Distinct Mitochondrial Proteins Including OXPHOS Components and Matrix Enzymes
Source: Int J Mol Sci. 2018 Mar 16;19(3):877. doi: 10.3390/ijms19030877 (PMC5877738; doi:10.3390/ijms19030877)
Supplement: Supplementary file 1 [file ijms-19-00877-s001.zip › Figure S2.pptx]

## Slide 1
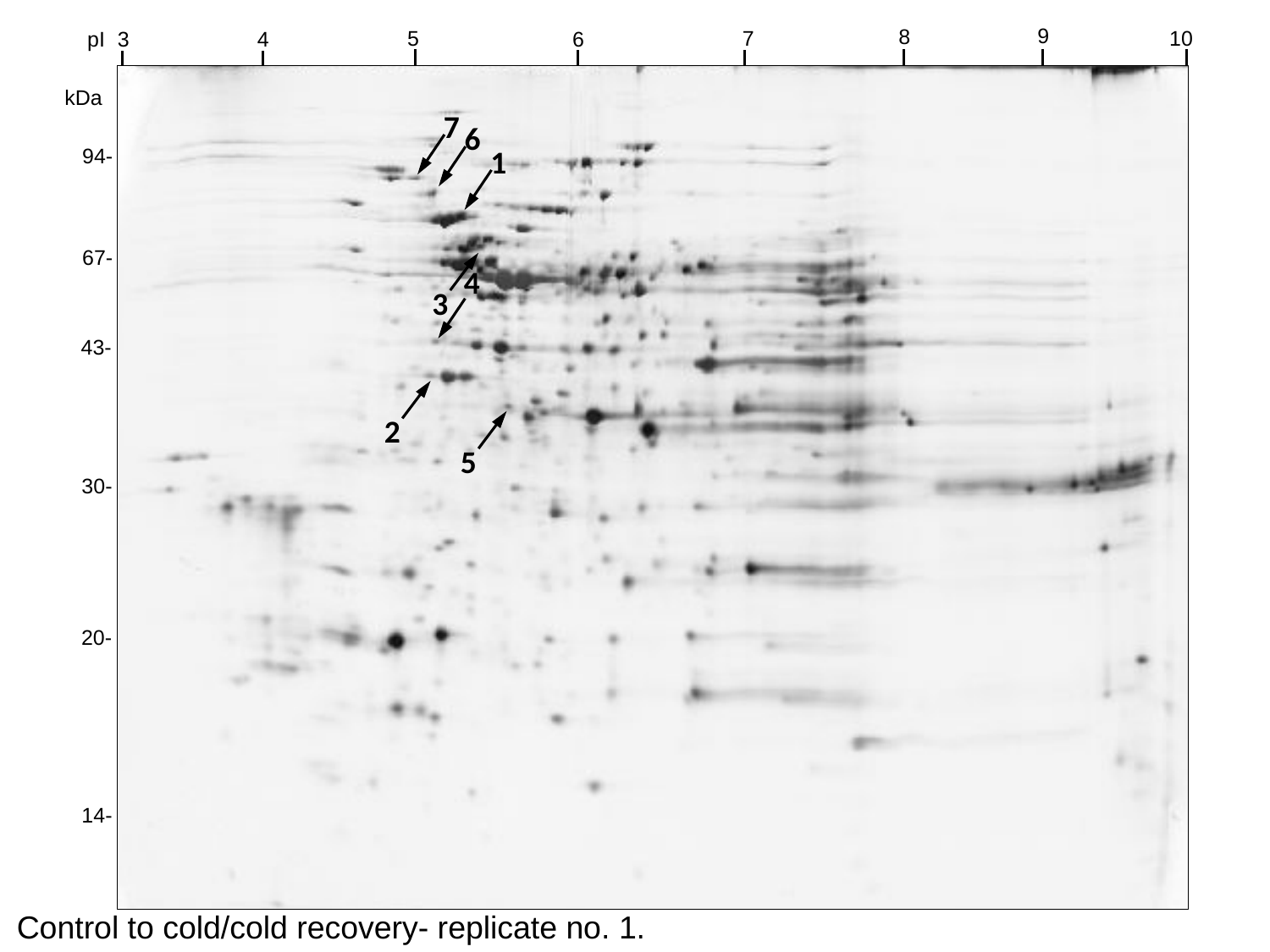

9
8
10
7
5
pI
3
4
6
kDa
7
6
1
94-
67-
4
3
43-
2
5
30-
20-
14-
Control to cold/cold recovery- replicate no. 1.

## Slide 2
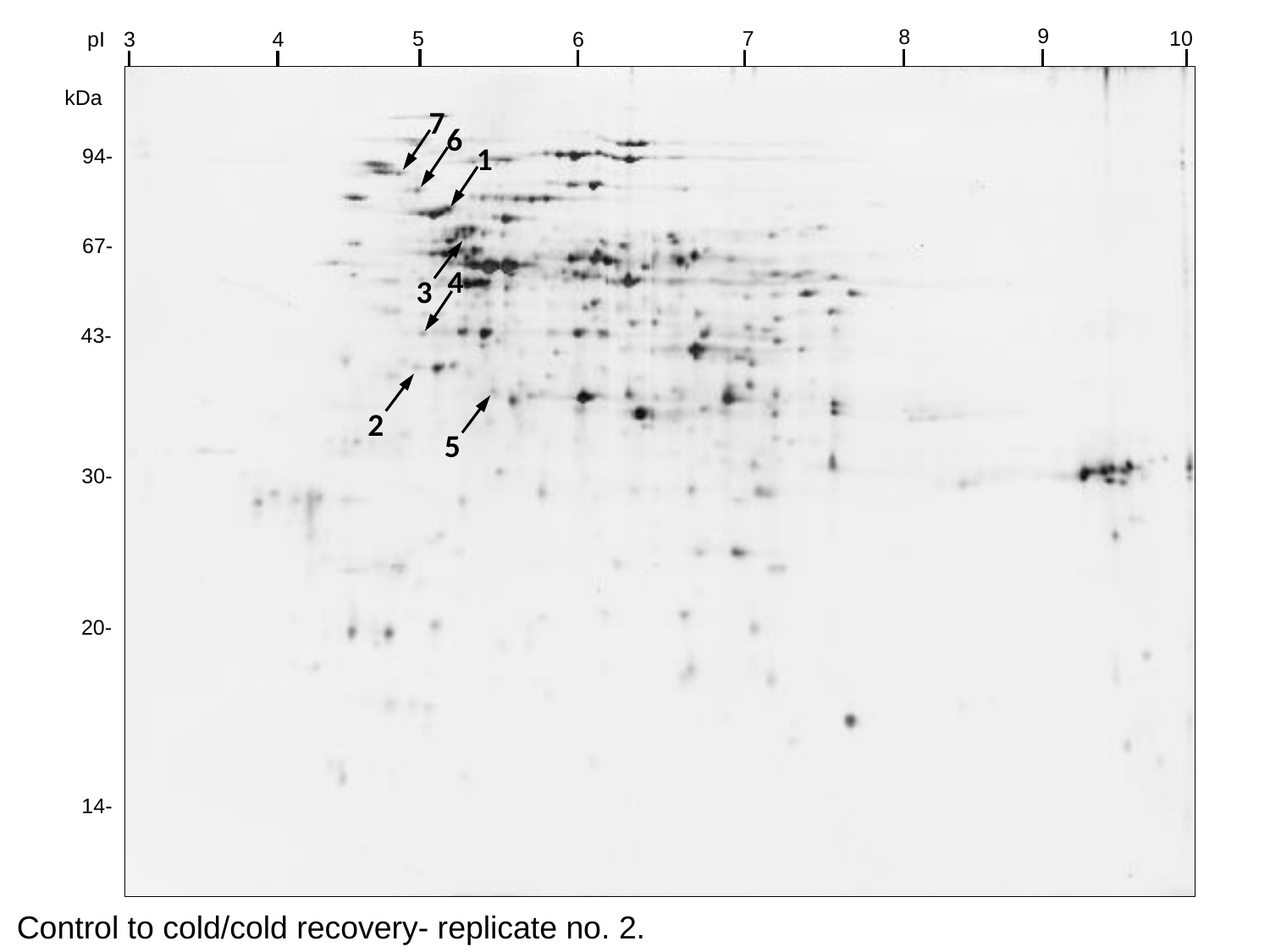

9
8
10
7
5
pI
3
4
6
kDa
7
6
1
94-
67-
4
3
43-
2
5
30-
20-
14-
Control to cold/cold recovery- replicate no. 2.

## Slide 3
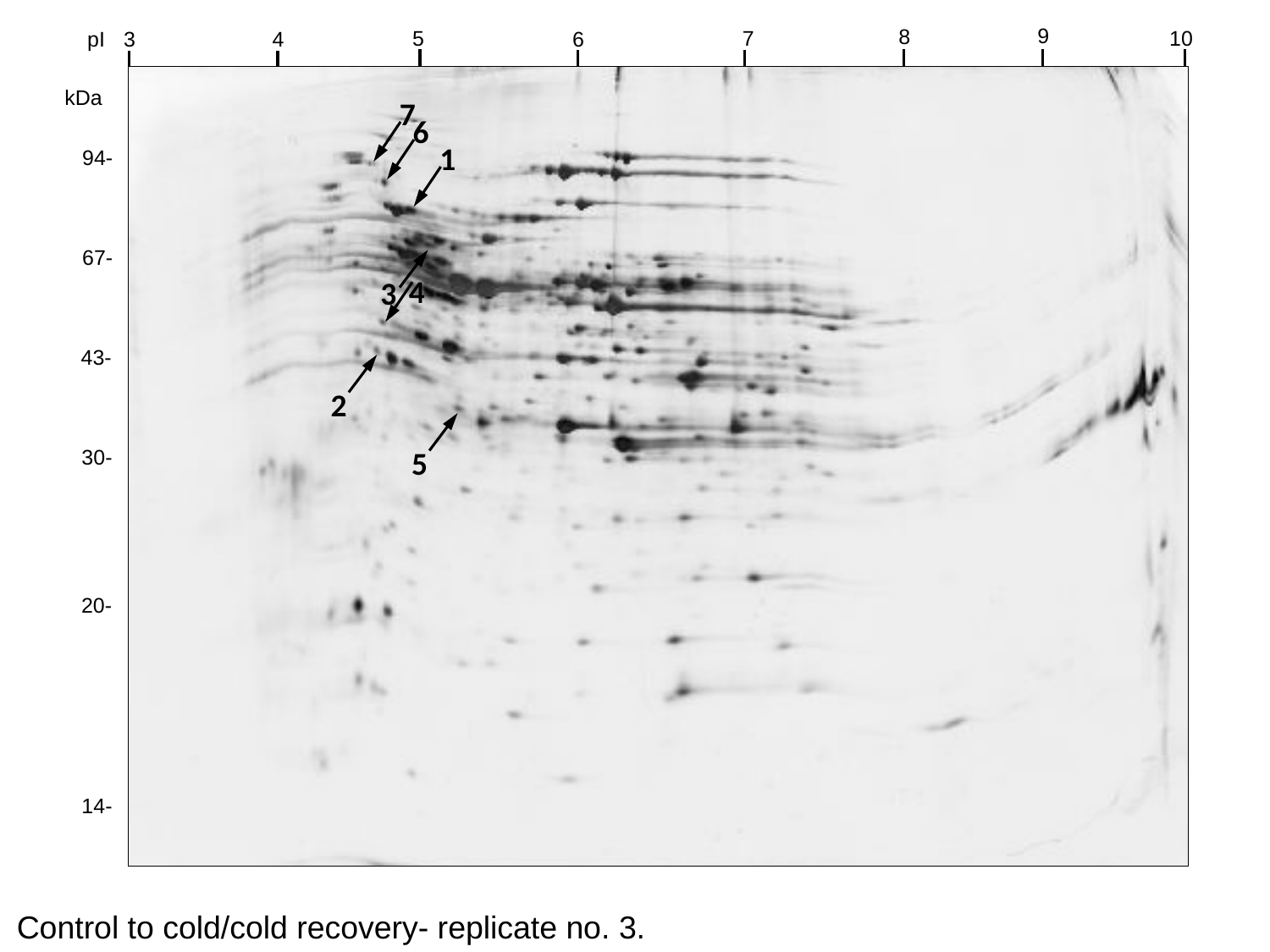

9
8
10
7
5
pI
3
4
6
kDa
7
6
1
94-
67-
4
3
43-
2
5
30-
20-
14-
Control to cold/cold recovery- replicate no. 3.

## Slide 4
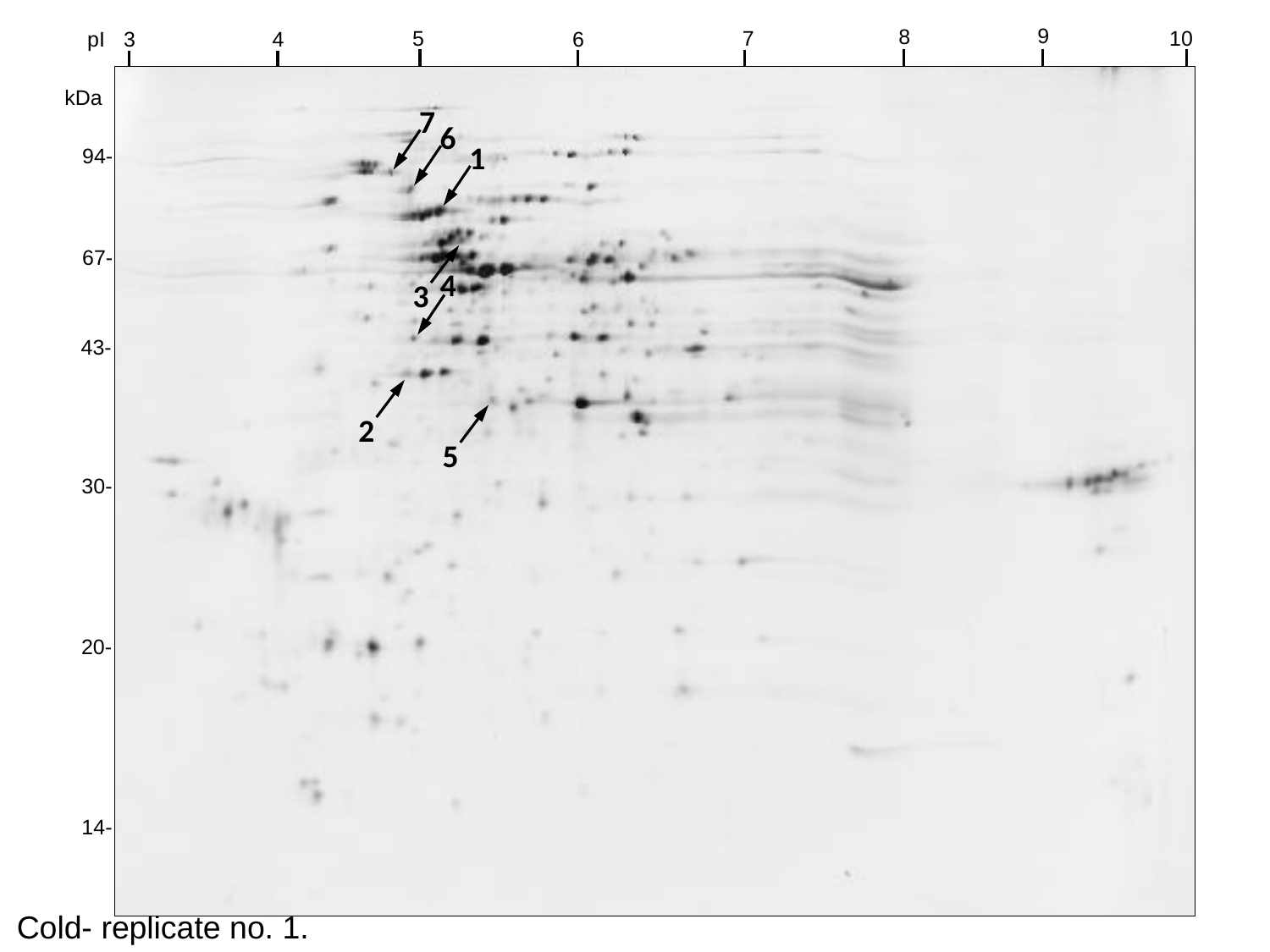

9
8
10
7
5
pI
3
4
6
kDa
7
6
1
94-
67-
4
3
43-
2
5
30-
20-
14-
Cold- replicate no. 1.

## Slide 5
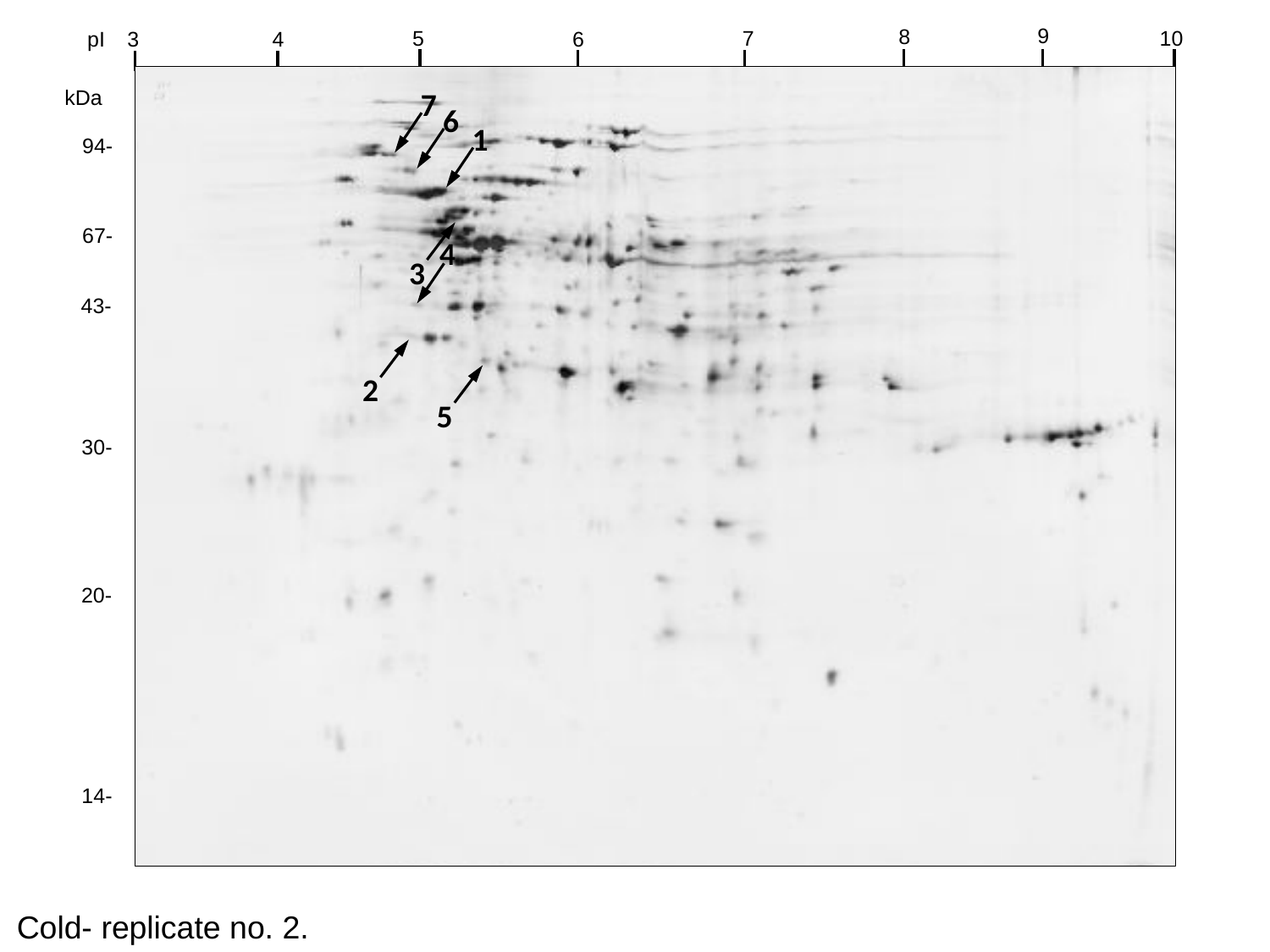

9
8
10
7
5
pI
3
4
6
kDa
7
6
1
94-
67-
4
3
43-
2
5
30-
20-
14-
Cold- replicate no. 2.

## Slide 6
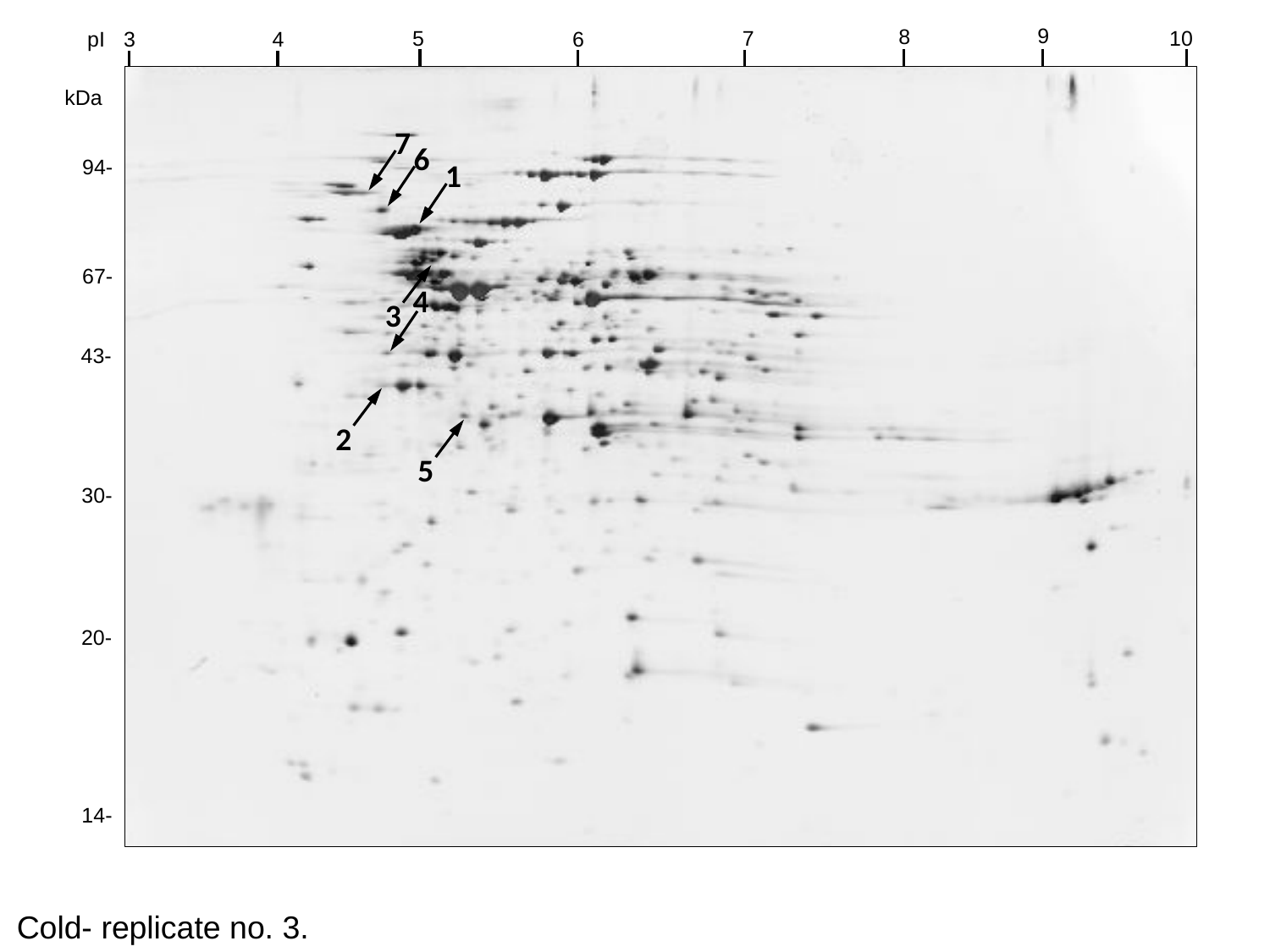

9
8
10
7
5
pI
3
4
6
kDa
7
6
94-
1
67-
4
3
43-
2
5
30-
20-
14-
Cold- replicate no. 3.

## Slide 7
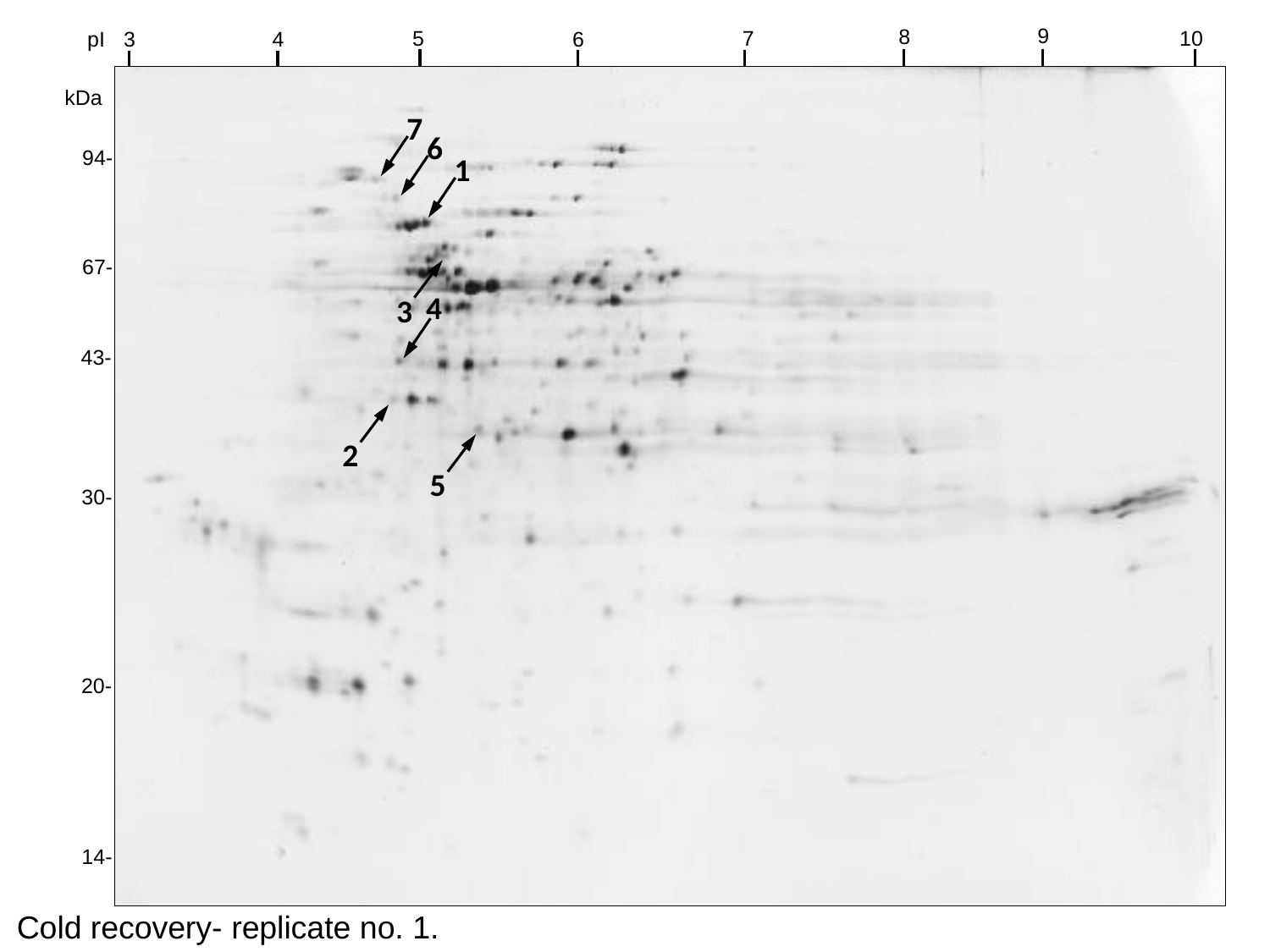

9
8
10
7
5
pI
3
4
6
kDa
7
6
94-
1
67-
4
3
43-
2
5
30-
20-
14-
Cold recovery- replicate no. 1.

## Slide 8
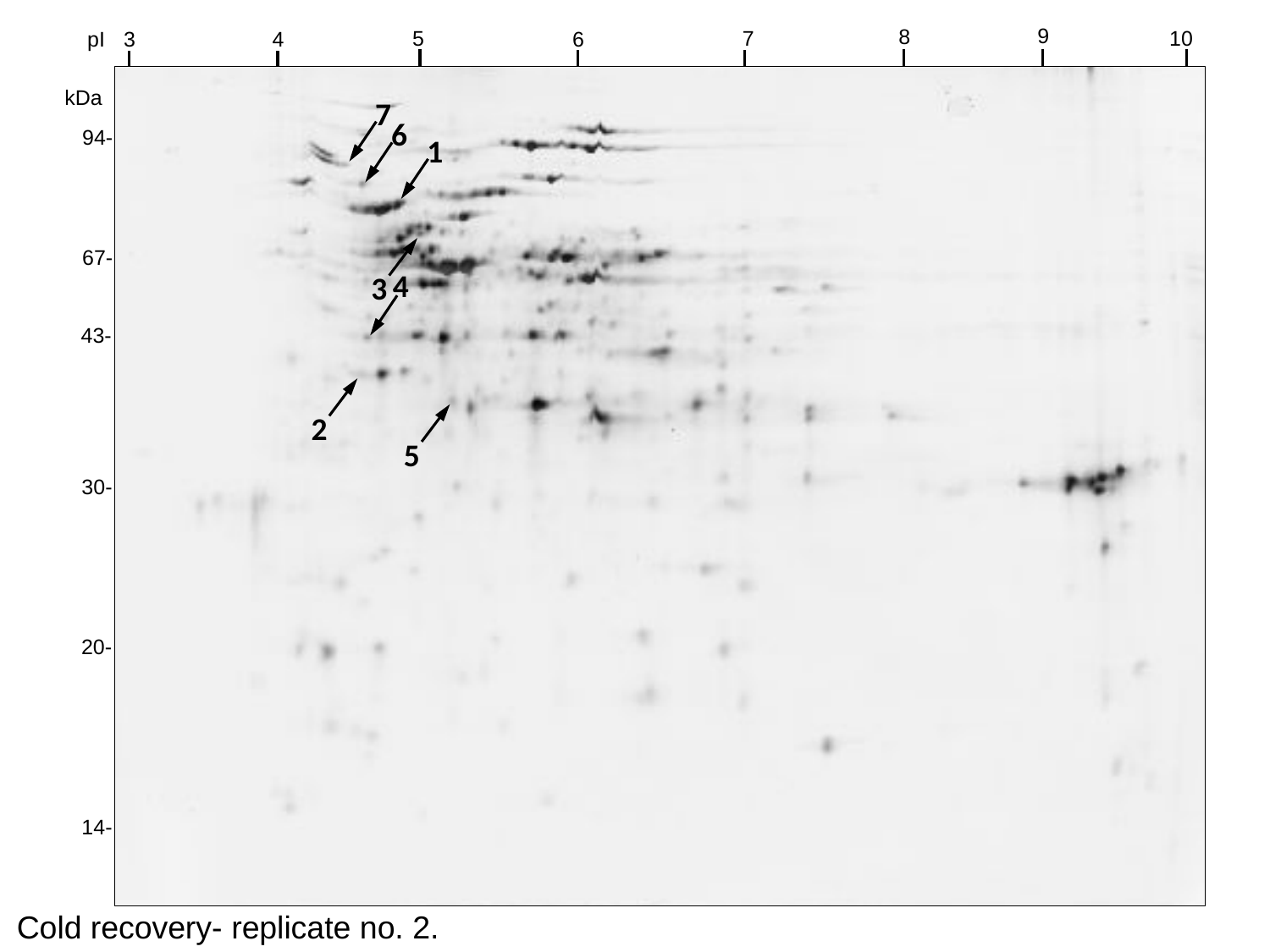

9
8
10
7
5
pI
3
4
6
kDa
7
6
94-
1
67-
4
3
43-
2
5
30-
20-
14-
Cold recovery- replicate no. 2.

## Slide 9
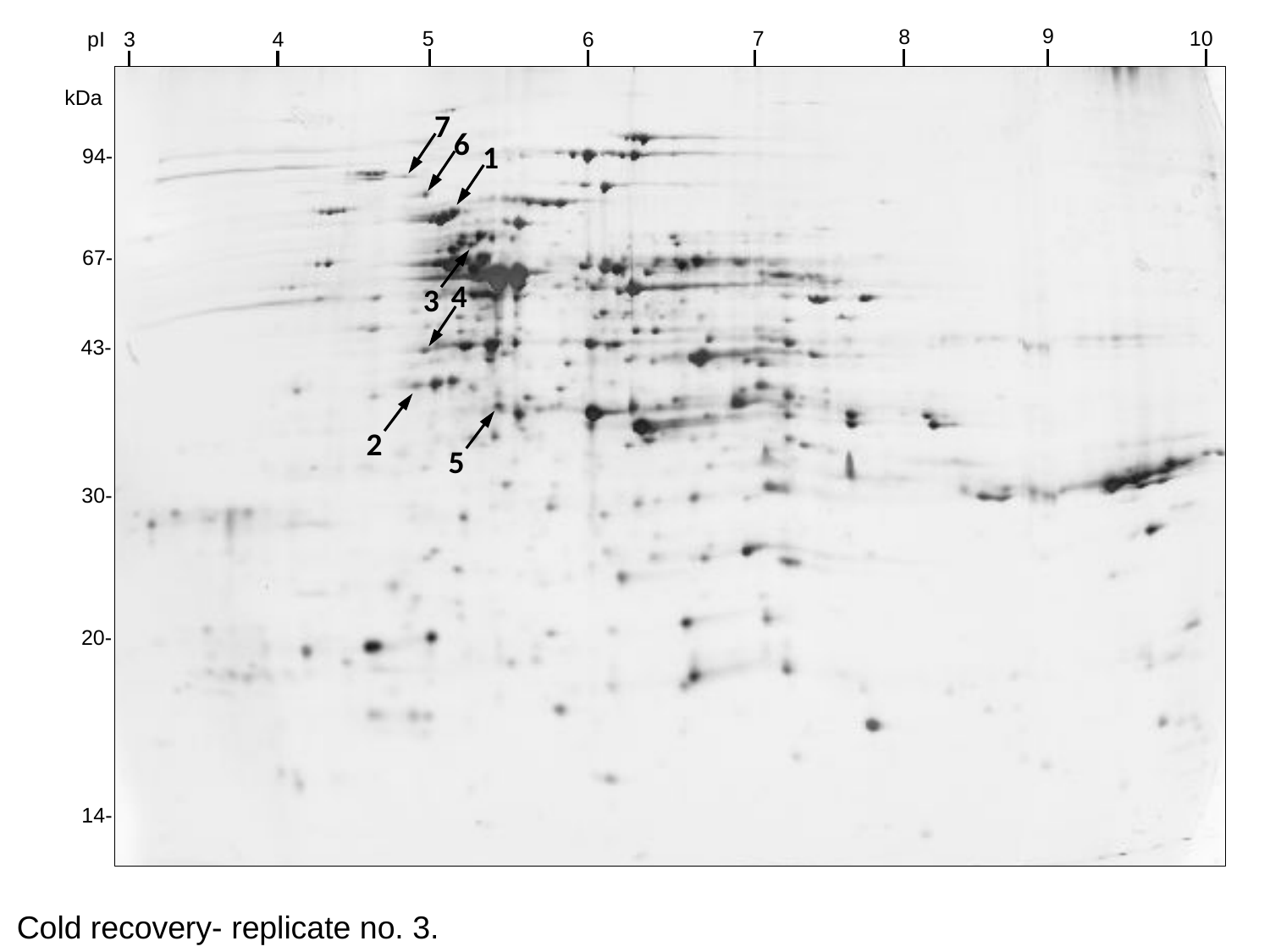

9
8
10
7
5
pI
3
4
6
kDa
7
6
1
94-
67-
4
3
43-
2
5
30-
20-
14-
Cold recovery- replicate no. 3.

## Slide 10
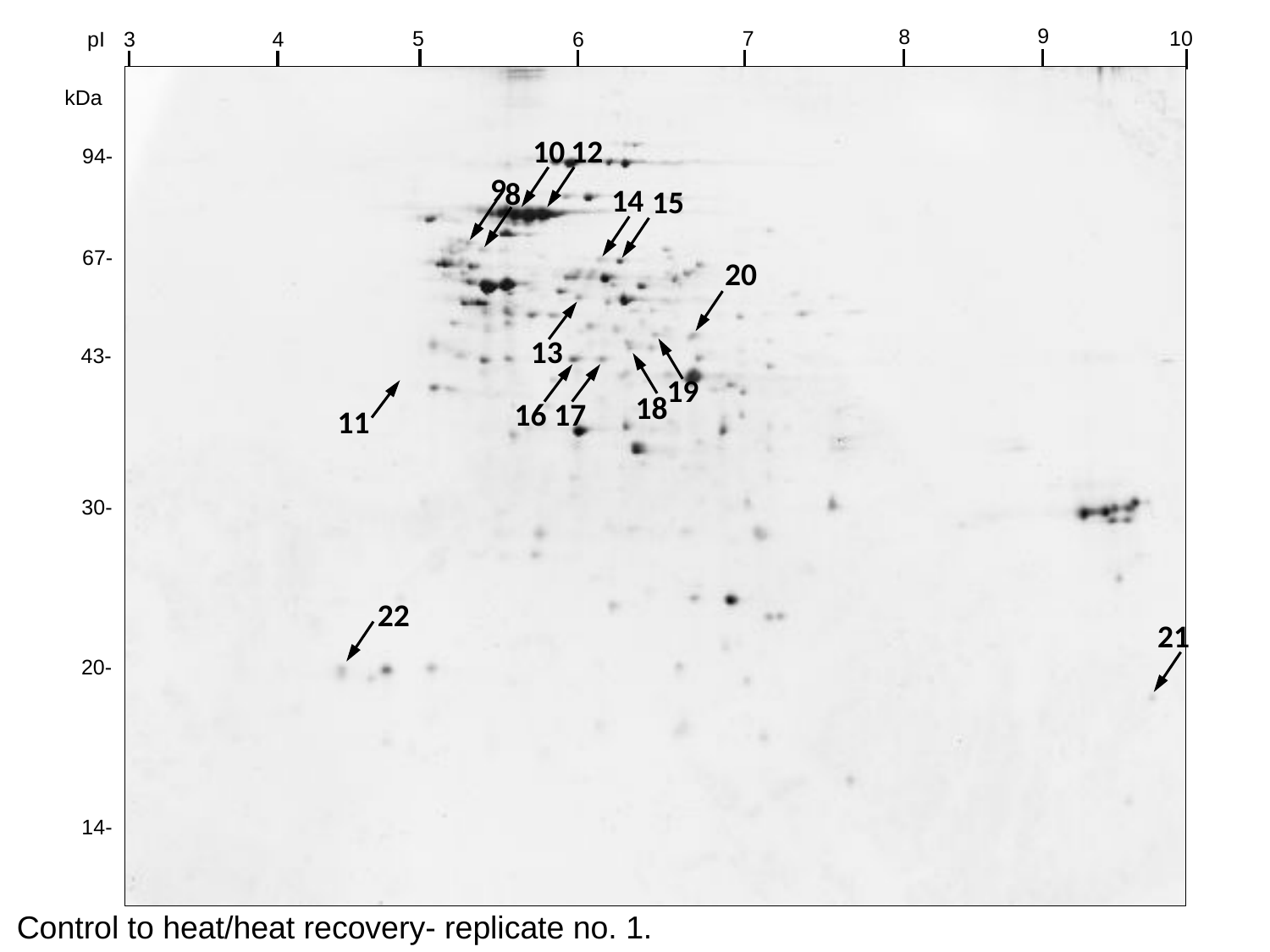

9
8
10
7
5
pI
3
4
6
kDa
12
10
94-
9
8
14
15
67-
20
13
43-
19
18
16
17
11
30-
22
21
20-
14-
Control to heat/heat recovery- replicate no. 1.

## Slide 11
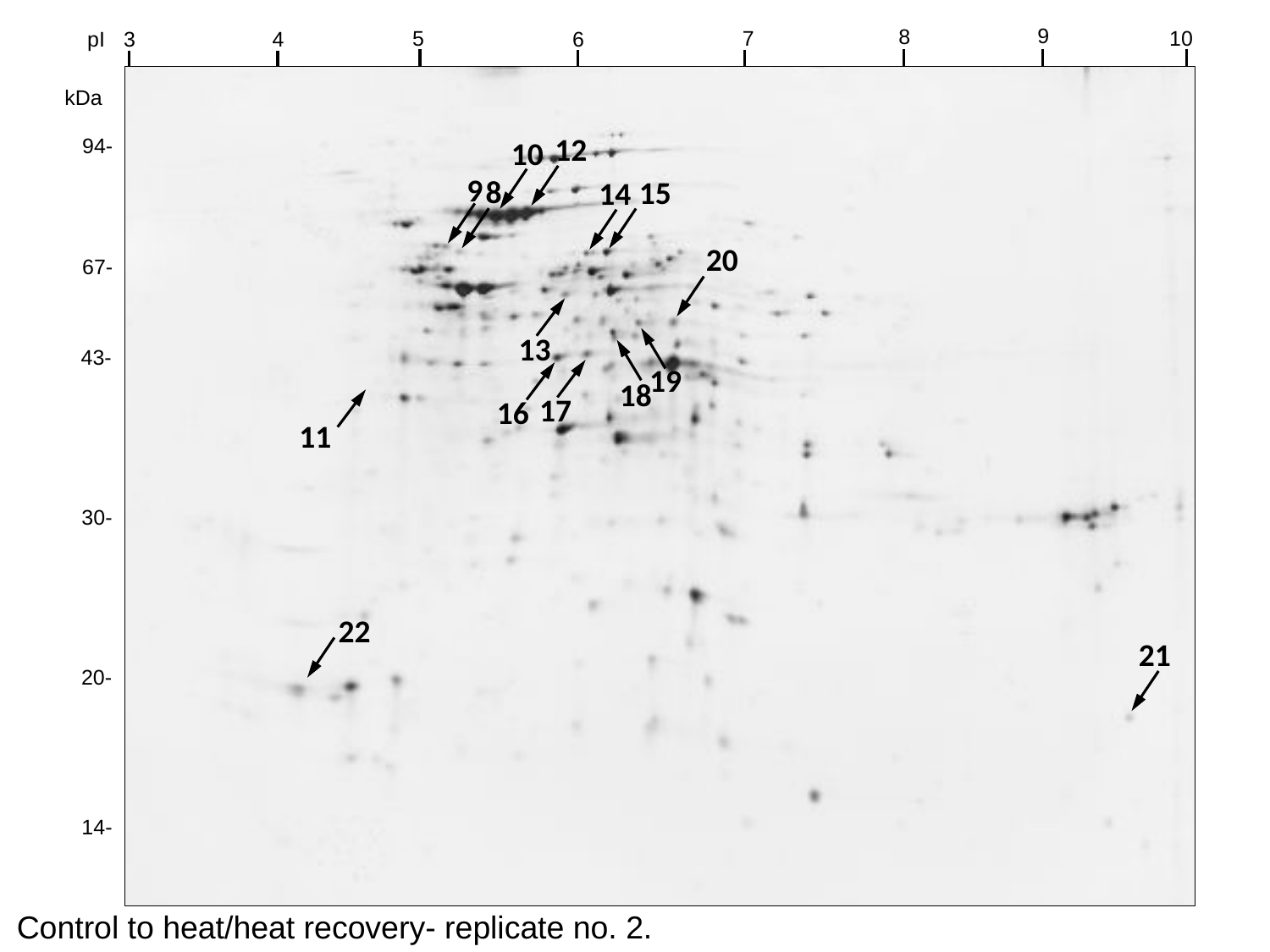

9
8
10
7
5
pI
3
4
6
kDa
12
94-
10
9
8
15
14
20
67-
13
43-
19
18
17
16
11
30-
22
21
20-
14-
Control to heat/heat recovery- replicate no. 2.

## Slide 12
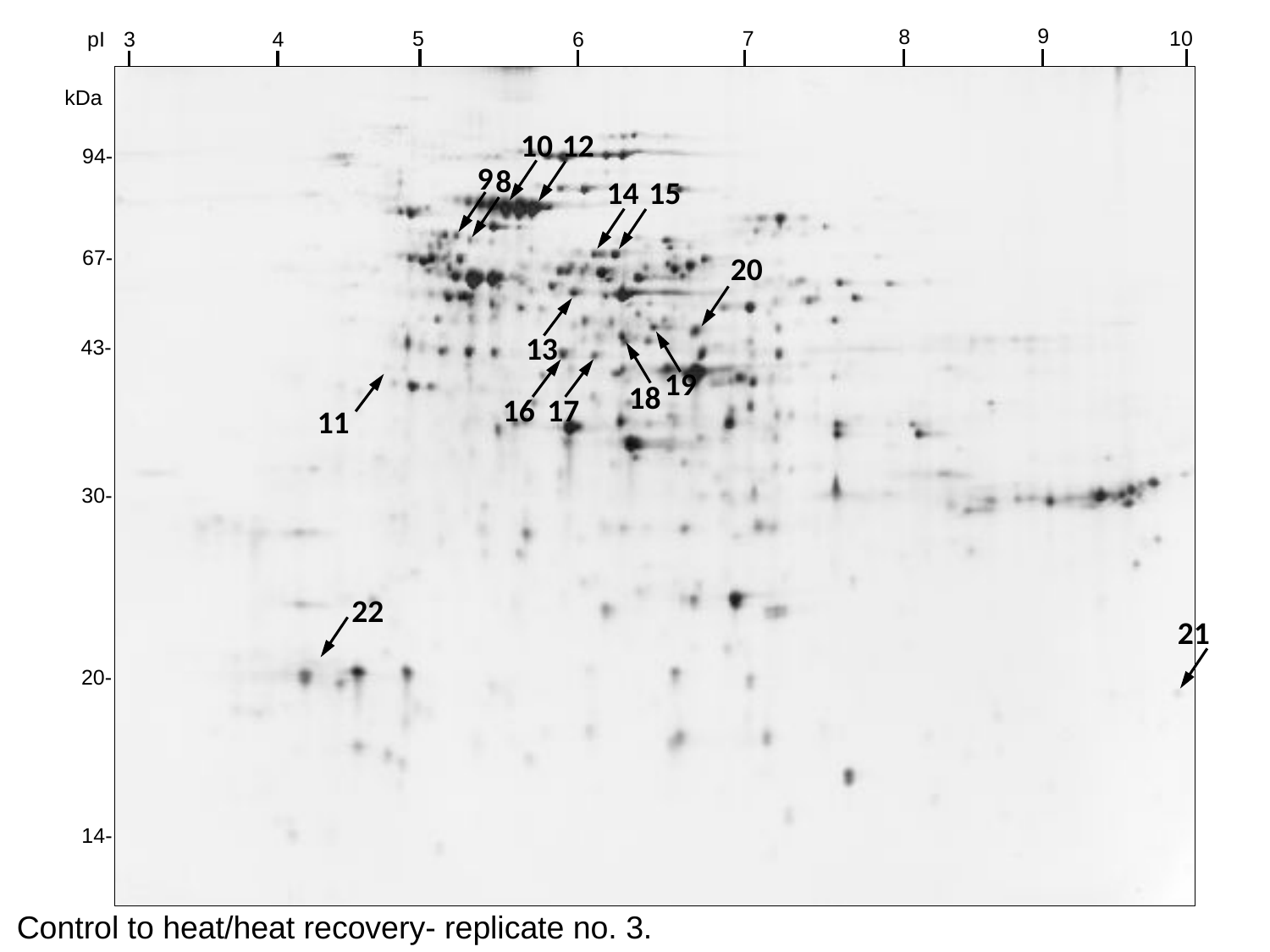

9
8
10
7
5
pI
3
4
6
kDa
10
12
94-
9
8
14
15
67-
20
13
43-
19
18
16
17
11
30-
22
21
20-
14-
Control to heat/heat recovery- replicate no. 3.

## Slide 13
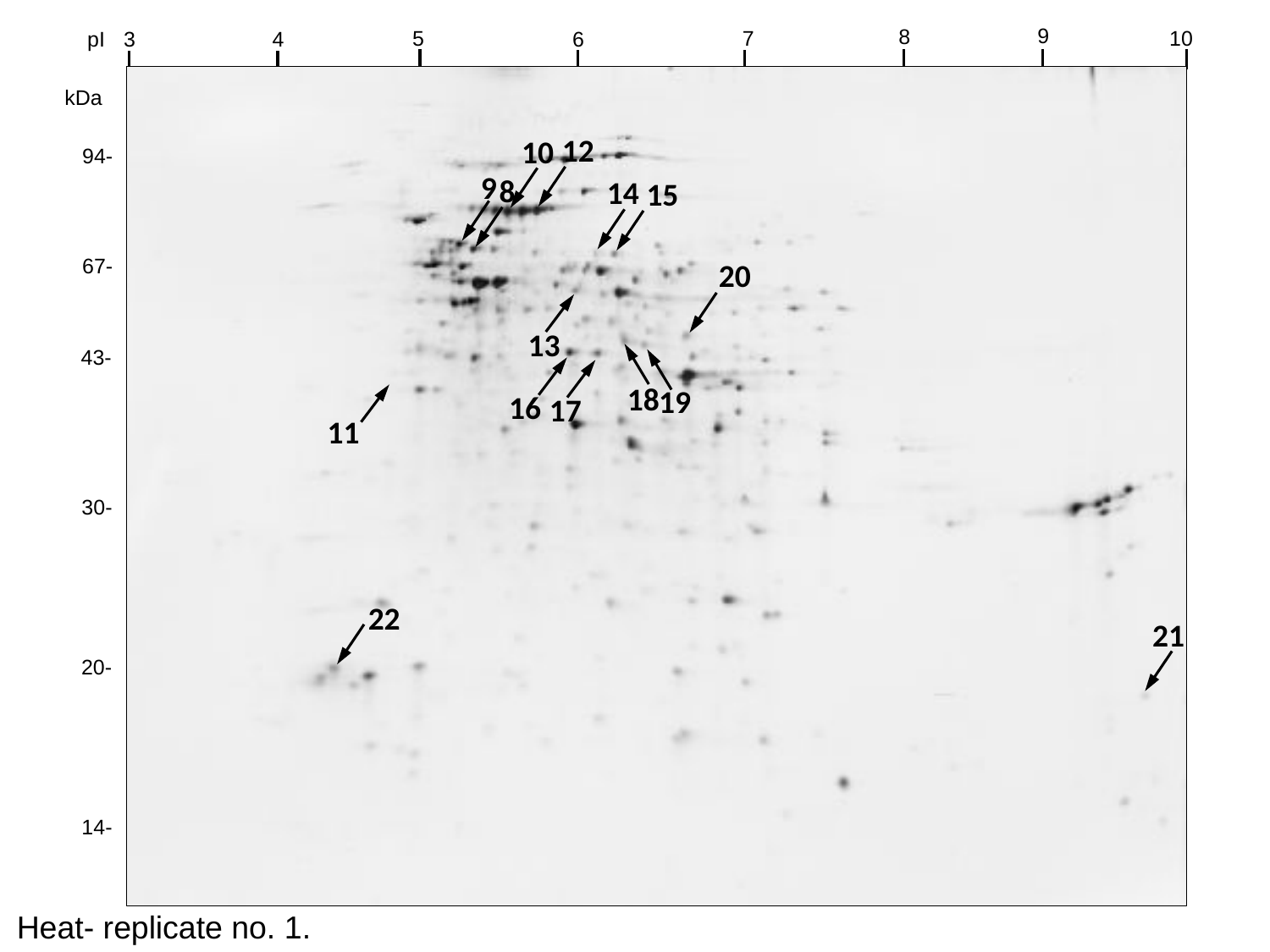

9
8
10
7
5
pI
3
4
6
kDa
12
10
94-
9
8
14
15
67-
20
13
43-
18
19
16
17
11
30-
22
21
20-
14-
Heat- replicate no. 1.

## Slide 14
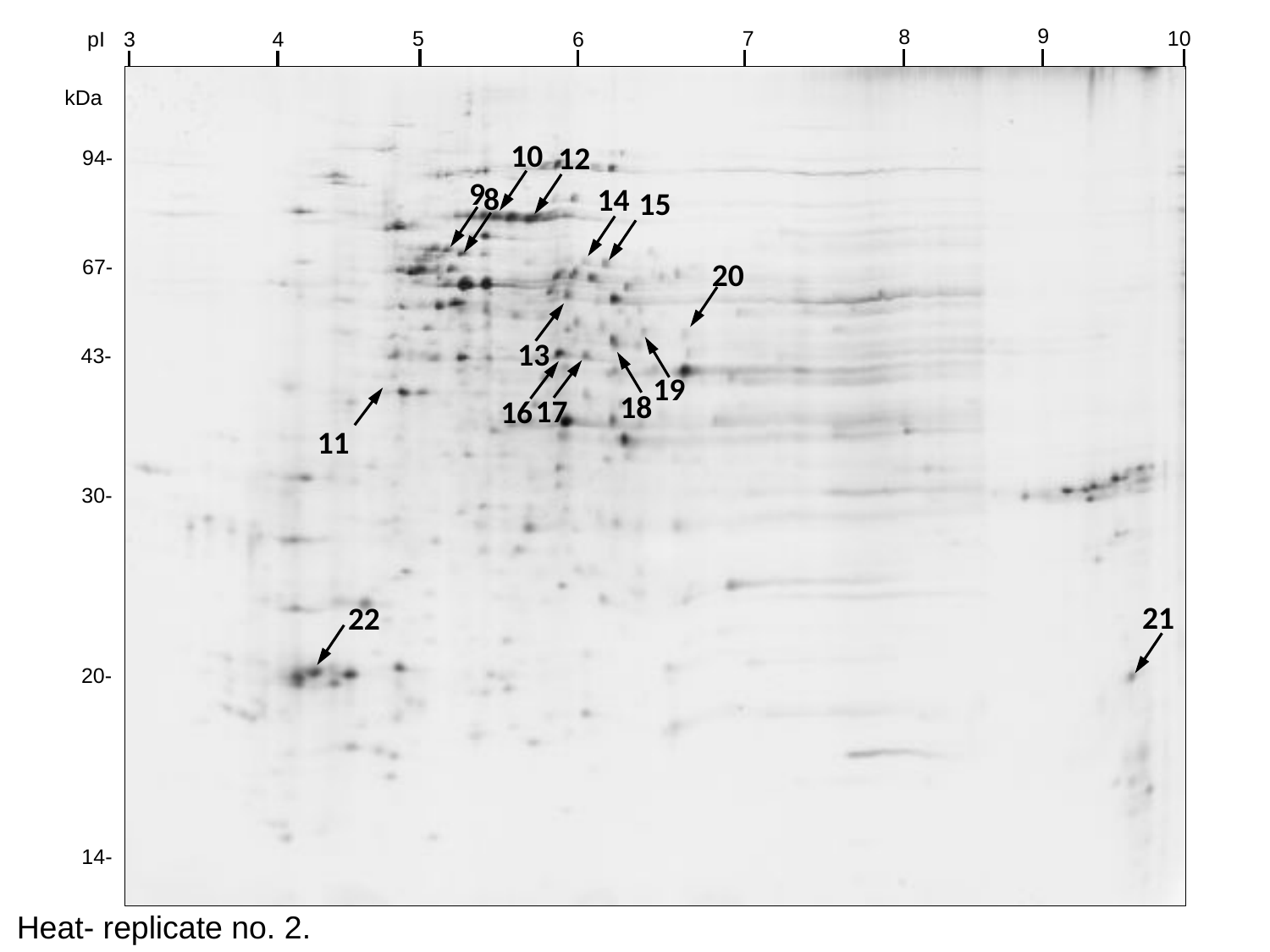

9
8
10
7
5
pI
3
4
6
kDa
10
12
94-
9
8
14
15
67-
20
13
43-
19
18
17
16
11
30-
21
22
20-
14-
Heat- replicate no. 2.

## Slide 15
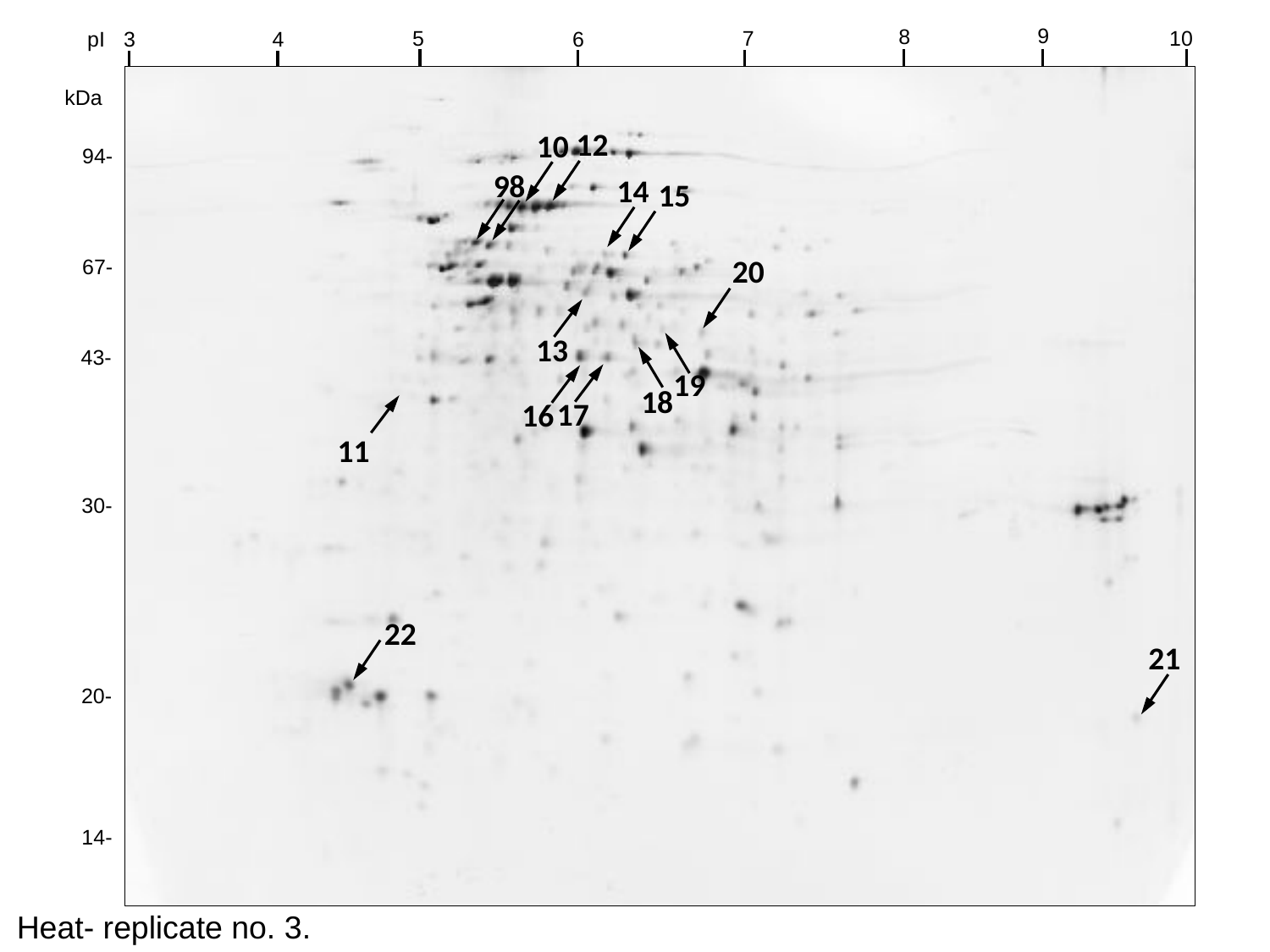

9
8
10
7
5
pI
3
4
6
kDa
12
10
94-
8
9
14
15
20
67-
13
43-
19
18
17
16
11
30-
22
21
20-
14-
Heat- replicate no. 3.

## Slide 16
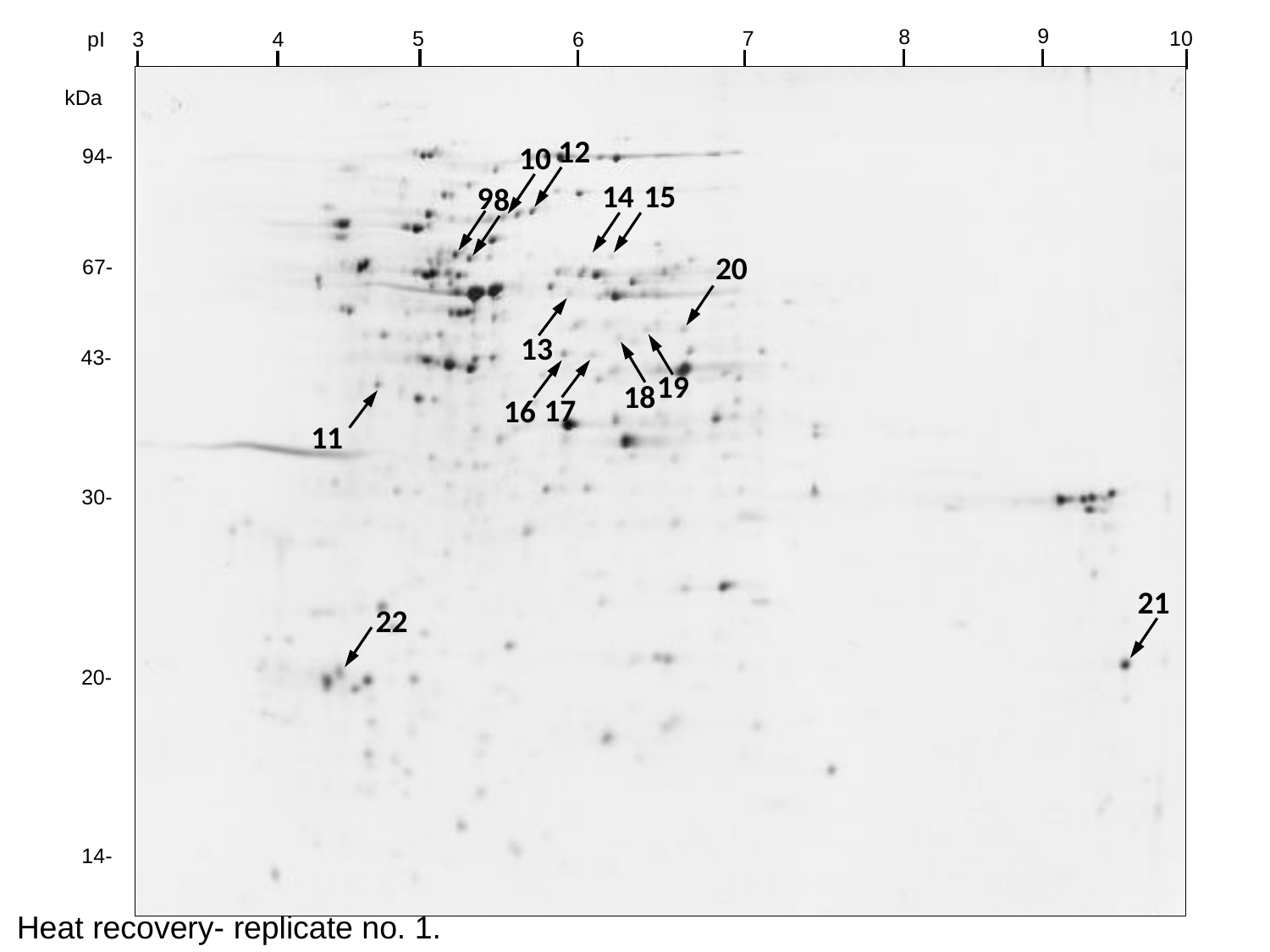

9
8
10
7
5
pI
3
4
6
kDa
12
10
94-
14
15
9
8
20
67-
13
43-
19
18
17
16
11
30-
21
22
20-
14-
Heat recovery- replicate no. 1.

## Slide 17
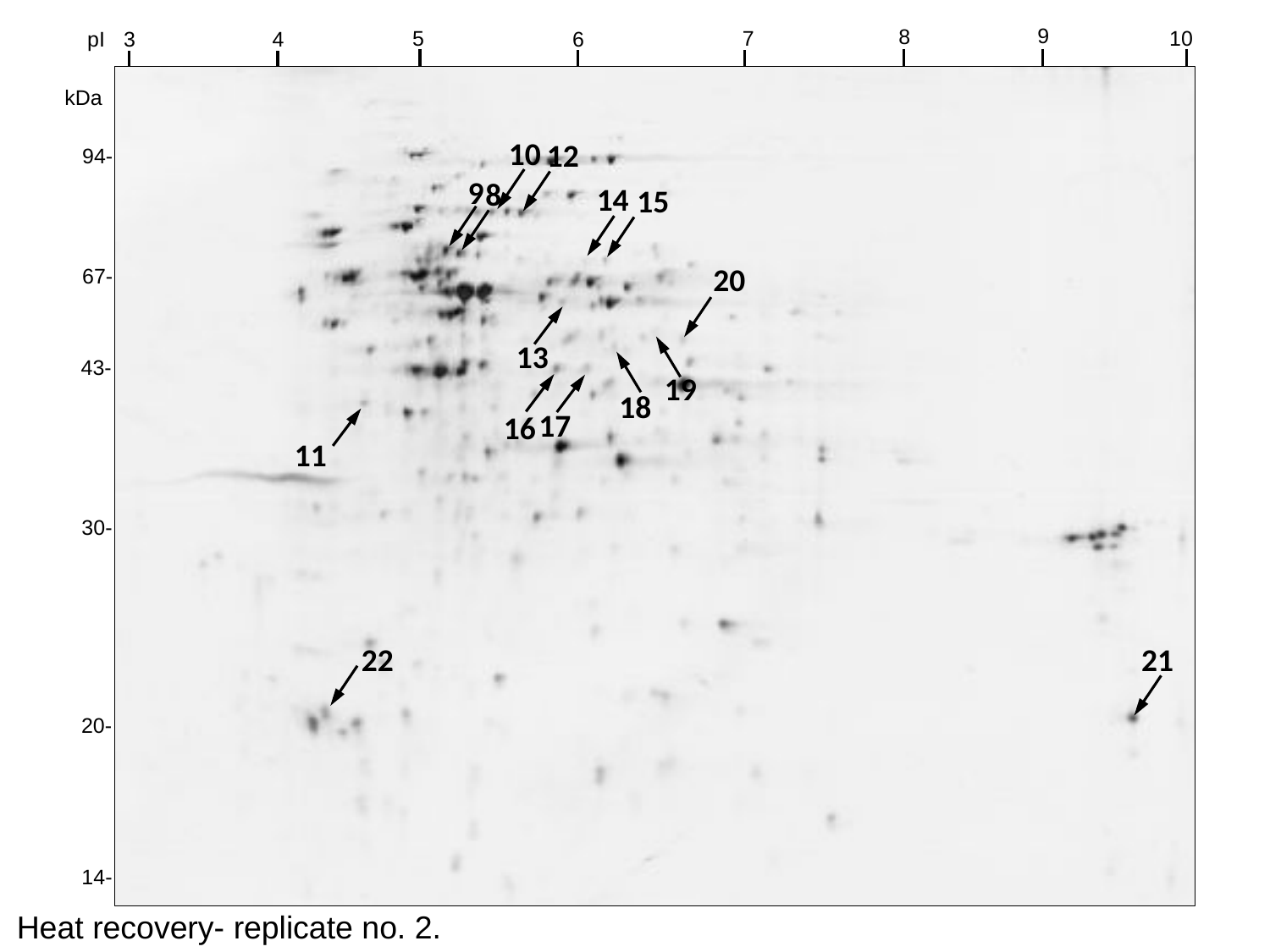

9
8
10
7
5
pI
3
4
6
kDa
10
12
94-
9
8
14
15
20
67-
13
43-
19
18
17
16
11
30-
22
21
20-
14-
Heat recovery- replicate no. 2.

## Slide 18
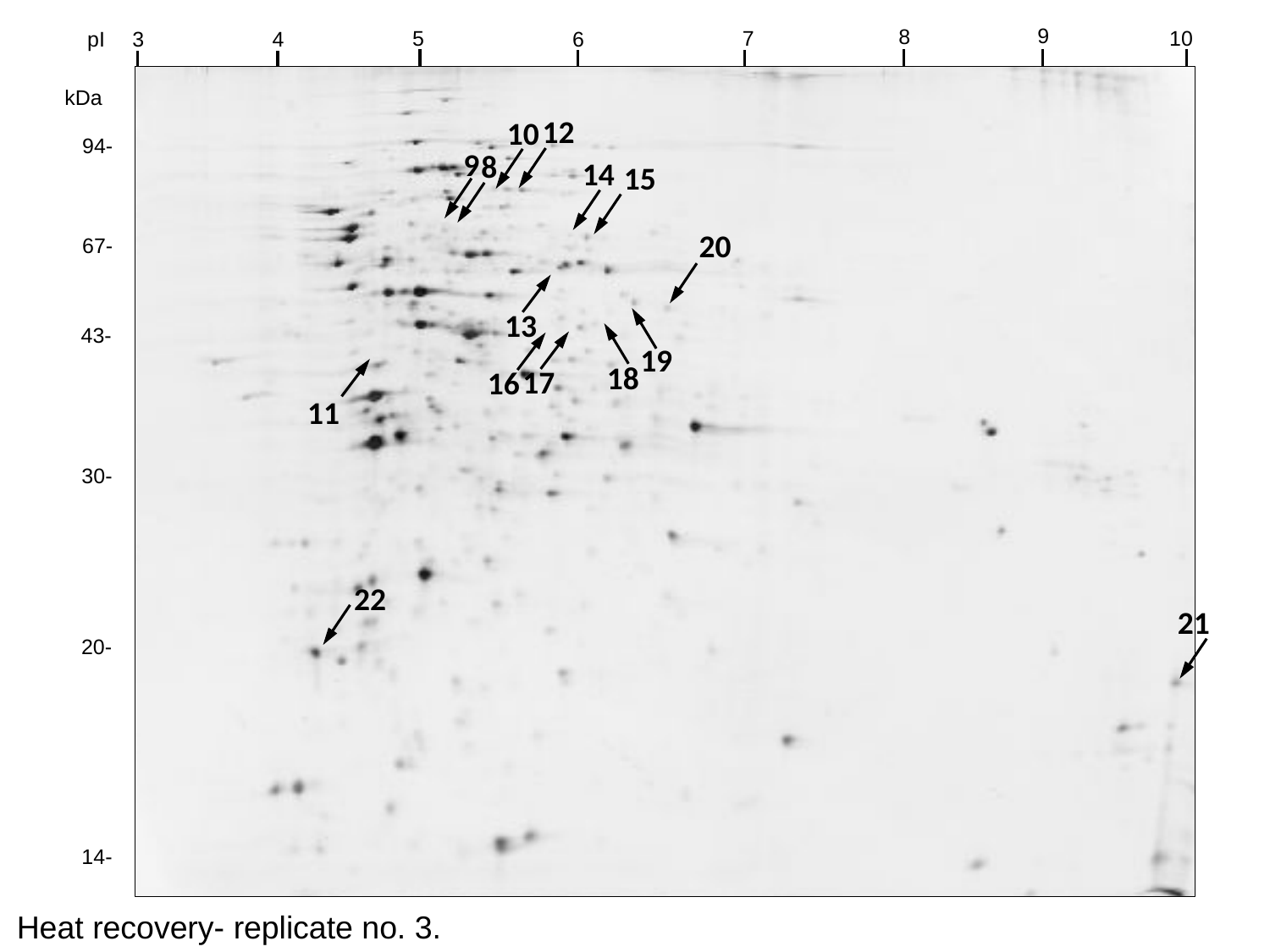

9
8
10
7
5
pI
3
4
6
kDa
12
10
94-
9
8
14
15
20
67-
13
43-
19
18
17
16
11
30-
22
21
20-
14-
Heat recovery- replicate no. 3.

## Slide 19
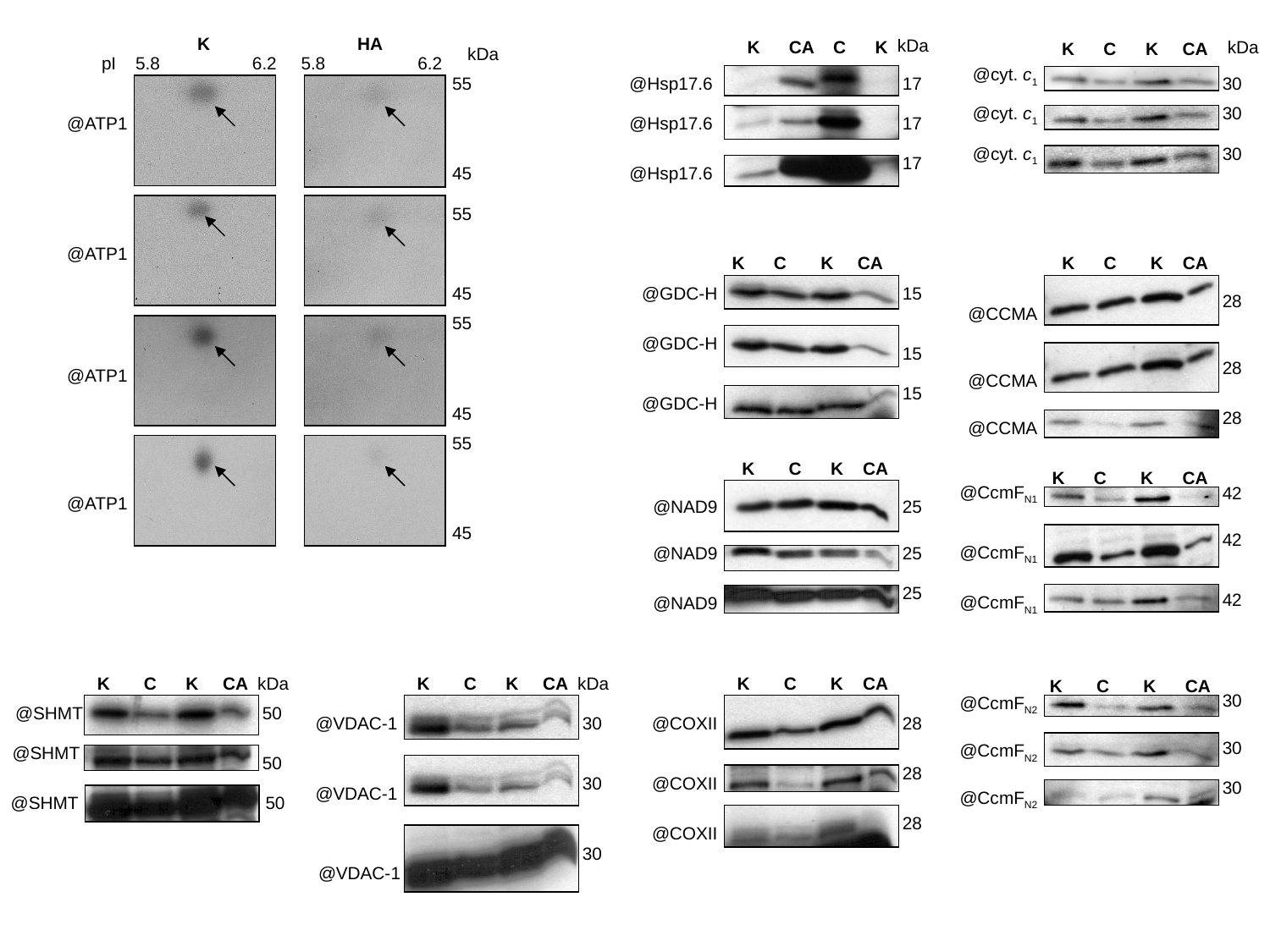

K
HA
kDa
kDa
 K CA C K
 K C K CA
kDa
 pI 5.8 6.2 5.8 6.2
@cyt. c1
 30
 55
@Hsp17.6
 17
@cyt. c1
 30
@Hsp17.6
 17
@ATP1
@cyt. c1
 30
 17
@Hsp17.6
 45
 55
@ATP1
 K C K CA
 K C K CA
 45
@GDC-H
 15
 28
@CCMA
 55
@GDC-H
 15
 28
@ATP1
@CCMA
 15
@GDC-H
 45
 28
@CCMA
 55
 K C K CA
 K C K CA
@CcmFN1
 42
@ATP1
@NAD9
 25
 45
 42
@CcmFN1
@NAD9
 25
 25
 42
@CcmFN1
@NAD9
 K C K CA
kDa
 K C K CA
kDa
 K C K CA
 K C K CA
 30
@CcmFN2
@SHMT
 50
@VDAC-1
 30
@COXII
 28
 30
@CcmFN2
@SHMT
 50
 28
@COXII
 30
 30
@VDAC-1
@CcmFN2
@SHMT
 50
 28
@COXII
 30
@VDAC-1

## Slide 20
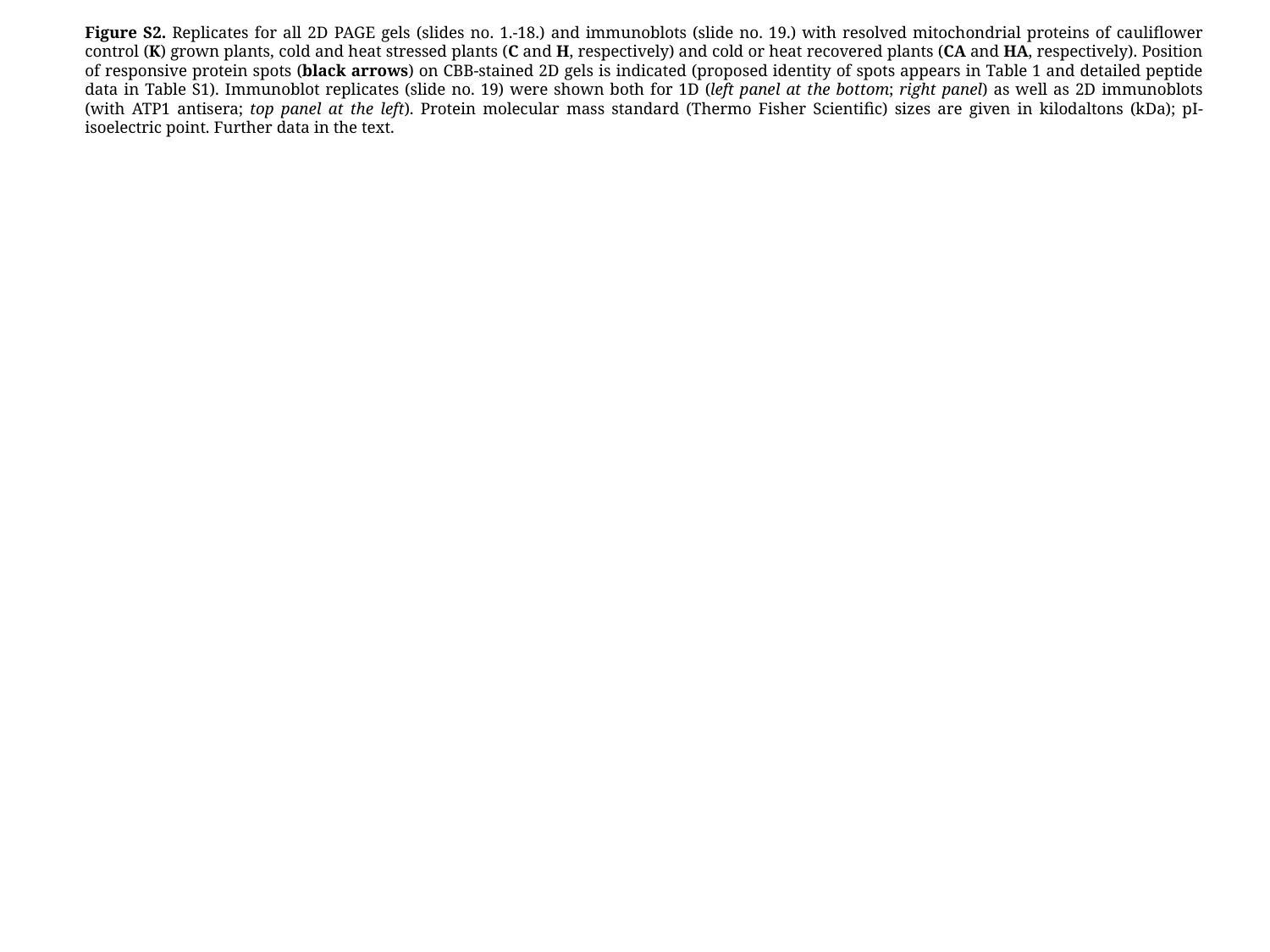

Figure S2. Replicates for all 2D PAGE gels (slides no. 1.-18.) and immunoblots (slide no. 19.) with resolved mitochondrial proteins of cauliflower control (K) grown plants, cold and heat stressed plants (C and H, respectively) and cold or heat recovered plants (CA and HA, respectively). Position of responsive protein spots (black arrows) on CBB-stained 2D gels is indicated (proposed identity of spots appears in Table 1 and detailed peptide data in Table S1). Immunoblot replicates (slide no. 19) were shown both for 1D (left panel at the bottom; right panel) as well as 2D immunoblots (with ATP1 antisera; top panel at the left). Protein molecular mass standard (Thermo Fisher Scientific) sizes are given in kilodaltons (kDa); pI- isoelectric point. Further data in the text.
