# Supplementary material for: Cold and Heat Stress Diversely Alter Both Cauliflower Respiration and Distinct Mitochondrial Proteins Including OXPHOS Components and Matrix Enzymes
Source: Int J Mol Sci. 2018 Mar 16;19(3):877. doi: 10.3390/ijms19030877 (PMC5877738; doi:10.3390/ijms19030877)
Supplement: Supplementary file 1 [file ijms-19-00877-s001.zip › Figure S1.doc]

**kDa**

**20**

**H**

**K_C_**

**HA**

**CA**

**C**

**K_H_**

**pI**

**3	4	5	6	7	8	9	10**

**pI**

**3	4	5	6	7	8	9	10**

**94**

**67**

**43**

**30**

**14**

**94**

**67**

**43**

**30**

**20**

**14**

**kDa**

**Figure S1.** Representative silver-stained 2D gels of cauliflower mitochondrial proteome variations from control (**K**) grown plants, cold- and heat- (**C** and **H**, respectively) stressed plants and from cold- or heat-recovered plants (**CA** and **HA**, respectively). Control for H and HA was denoted as **K_H_** and the one for C and CA variants- as **K_C_**. 100 μg of mitochondrial proteins were loaded onto all gels. 24- cm immobilized pH gradient strips (linear pH 3-10) for the first dimension and precasted Ettan DALT 12.5% SDS- polyacrylamide gels for the second dimension were used. Individual spots with variable intensity in each variant are indicated by green arrows next to the boxes. Protein molecular mass standard (Thermo Fisher Scientific) sizes are given in kilodaltons (kDa). pI- isoelectric point
