# Supplementary material for: Cold and Heat Stress Diversely Alter Both Cauliflower Respiration and Distinct Mitochondrial Proteins Including OXPHOS Components and Matrix Enzymes
Source: Int J Mol Sci. 2018 Mar 16;19(3):877. doi: 10.3390/ijms19030877 (PMC5877738; doi:10.3390/ijms19030877)
Supplement: Supplementary file 1 [file ijms-19-00877-s001.zip › Table S5.doc]

**Table S5.** Primers used for RT-PCR analyses.

| **Primer identifier** | **Primer sequence** | **Primer length** | **Primer T*_m_* [^o^C]** | **T*_an_***  **[^o^C]** | **Primer positions and accession.version identifiers**  **of respective templates** | | **Amplicon description** |
| --- | --- | --- | --- | --- | --- | --- | --- |
| NAD9-F | ATGGATAACCAATTCATTTTCAAATA | 26-mer | 49.6 | 55 | 1-26 | DQ257689.1 | *nad9* (mitochondrial CI subunit 9) |
| NAD9-R | TTATCCGTCGCTACGCTGTTCCCAAG | 26-mer | 64.7 |  | 573-548 |  |  |
| NAD6-F | CTTTCTGTTTTGTCGAGCCCTG | 22-mer | 58.1 | 55 | 7-28 | DQ288846.1 | *nad6* (mitochondrial CI subunit 6) |
| NAD6-R | TCGTACCTTTCCGGCATAAACC | 22-mer | 59.1 |  | 396-375 |  |  |
| NAD3-F | CTTCGCCAGCAAGGAGCGAG | 20-mer | 63.7 | 55 | 1501-1520 | DQ219817.1 | *nad3* (mitochondrial CI subunit 3) |
| NAD3-R | TACTCCCAATCCAAAGCACCCCT | 23-mer | 62.2 |  | 1897-1875 |  |  |
| COX2-F | CAGTTCCGATGAGCAGTCACTC | 22-mer | 59.4 | 58 | 496-517 | AF036386.1 | *cox2* (mitochondrial CIV subunit 2) |
| COX2-R | CCCCGGTTTGGGGGATTAATTG | 22-mer | 60.7 |  | 866-845 |  |  |
| ATP1-F | AAGTGGATGAGATCGGTCGAGT | 22-mer | 59.1 | 55 | 91393-91414 | KJ820683.1 | *atp1* (mitochondrial ATP synthase subunit 1) |
| ATP1-R | TAGGCTCGTGCACTGATTTACG | 22-mer | 58.6 |  | 91758-91737 |  |  |
| ATP2-F | AGGTGTCTCATCACTTGGGTCA | 22-mer | 59.2 | 55 | 401- 422 | XM_009132853.2 | *ATP2* (mitochondrial ATP synthase subunit 2) |
| ATP2-R | ACACAGAGAAACCACCATGAGC | 22-mer | 58.8 |  | 805- 784 |  |  |
| HSP70-F | GCCTGTGGGGAATGATGTTATCGG | 24-mer | 62.1 | 55 | 196-219 | XM_013773775.1 | *HSP70* (mitochondrial heat shock 70 kDa protein) |
| HSP70-R | GGCTCGTTGATGATTCTCTGGACA | 24-mer | 60.8 |  | 717-694 |  |  |
| HSP81-F | TCCGTCGTGTCTTCATCATGGAC | 23-mer | 60.7 | 55 | 1457-1479 | NM_124985.5 | *HSP81* (mitochondrial heat shock 81 kDa protein) |
| HSP81-R | TCATCACCGCTCTTGGTCGAGTG | 23-mer | 63.1 |  | 1781-1759 |  |  |
| HSP17.6-F | CAAACGTGTTCGACCCATTCTC | 22-mer | 58.1 | 55 | 106-127 | AF022217.1 | *HSP17.6* (small heat shock protein) |
| HSP17.6-R | CCGGCAACTTAAACCTCCTCAT | 22-mer | 58.9 |  | 426-405 |  |  |
| CPN10-F | TGATGAAGCGTCTGATCCCAA | 21-mer | 57.8 | 50 | 92- 112 | XM_013881536.2 | *CPN10* (mitochondrial chaperonin 10) |
| CPN10-R | CCACTCGTAGCTACTTAGCCTT | 22-mer | 57.5 |  | 410-389 |  |  |
| PDH-E1-F | CAGCCTGGTACTGCCTCAGTTCC | 23-mer | 64.2 | 55 | 563- 584 | XM_013785321.2 | *PDH-E1* (mitochondrial pyruvate dehydrogenase E1 component) |
| PDH-E1-R | ACCGGTGCATCCAAGTATGAAAAG | 24-mer | 59.9 |  | 1037- 1014 |  |  |
| SCLβ-F | TGCTTTCCGTCAGAAAGAGATTTTT | 25-mer | 56.3 | 55 | 859- 883 | XM_013820521.2 | *SCLβ* (mitochondrial succinyl- CoA ligase subunit β) |
| SCLβ-R | TCCCTGCTCCACATTTGTTCCTTC | 24-mer | 61.3 |  | 1264-1241 |  |  |
| 3-PGDH-F | CGTGTCATTGCTCATGATCCCTATG | 25-mer | 59.1 | 55 | 945- 969 | XM_013797132.2 | *3-PGDH* (3-phosphoglycerate dehydrogenase) |
| 3-PGDH-R | CAACAGCTTCAGCAATTTCAATGG | 24-mer | 56.6 |  | 1350-1327 |  |  |
| MPPβ-F | CTGCTCAGAATATCAAGTCTATCAC | 25-mer | 53.9 | 55 | 852-876 | XM_013844876.2 | *MPPβ* (mitochondrial processing peptidase subunit β) |
| MPPβ-R | TCCACCACCAACATTCTTGTTCCAT | 25-mer | 60.0 |  | 1207-1184 |  |  |
| PRODH-F1 | CCCAAAACCAAACGAAGCTCAC | 22-mer | 58.9 | 55 | 282-303 | XM_013837689.1 | *PRODH* (mitochondrial proline dehydrogenase 1)  *P5CDH* (mitochondrial Δ-1-pyrroline-5-carboxylate dehydrogenase 12A1) |
| PRODH-R1 | AGGGCTTGAATCGGAGAAAACC | 22-mer | 59.5 |  | 840-819 |  |  |
| P5CDH-F1 | TGCAGGGAAAGTGGATAGGATCT | 23-mer | 59.2 | 55 | 315-337 | XM_013773859.1 |  |
| P5CDH-R1 | GGCCAACGGTAGCCATGACTTTG | 23-mer | 63.0 |  | 732-710 |  |  |
| ACT1-F1 | AAAATGGCTGAGGCTGATGAC | 21-mer | 57.1 | 55 | 1-21 | KC631780.1 | *ACT1* (actin 1) |
| ACT1-R1 | CTCACAACACCATGCTCAATAG | 22-mer | 54.7 |  | 239-218 |  |  |

Primer sequences are given in 5’🡪3’ orientation. The term ‘template’ refers to the accession used for primer design with their nucleotide coordinates indicating primer position. F, forward (upper) primer; R, reverse (lower) primer; T*_m_*, melting temperature (calculated according to ThermoScientific T*_m_* Calculator at [www.thermofisher.com](../../Downloads/www.thermofisher.com)); T*_an_*, annealing temperature in RT-PCR assays. T*_m_* and T*_an_* values are given at Celsius degrees (^o^C).
