# Supplementary material for: Cold and Heat Stress Diversely Alter Both Cauliflower Respiration and Distinct Mitochondrial Proteins Including OXPHOS Components and Matrix Enzymes
Source: Int J Mol Sci. 2018 Mar 16;19(3):877. doi: 10.3390/ijms19030877 (PMC5877738; doi:10.3390/ijms19030877)
Supplement: Supplementary file 1 [file ijms-19-00877-s001.zip › Table S4.doc]

**Table S4.** List of antibodies used in the study.

| **Antibody** | **Protein** | **Origin** | **Serum** | **Dilution** | **Reference or source** |
| --- | --- | --- | --- | --- | --- |
| NAD9 | NADH dehydrogenase subunit 9 | *Triticum* ssp. | Rabbit (polyclonal) | 1/50,000 | Lamattina *et al*., 1993 |
| cyt. *c*_1_ | Cytochrome *c*_1_ | *Saccharomyces cerevisiae* | Rabbit (polyclonal) | 1/20,000 | gift of Prof. Gottfried Schatz (University of Basel) |
| ATP1 | Mitochondrial ATP synthase subunit α | *Saccharomyces cerevisiae* | Rabbit (polyclonal) | 1/50,000 | gift of Prof. Gottfried Schatz (University of Basel) |
| COXII | Cytochrome *c* oxidase subunit 2 | KLH-conjugated peptide | Rabbit (polyclonal) | 1/1,000 | Agrisera (Vännäs, Sweden), AS04 053A |
| VDAC-1 | Voltage-dependent anion channel 1 | KLH-conjugated peptide | Rabbit (polyclonal) | 1/1,000 | Agrisera (Vännäs, Sweden), AS07 212 |
| Hsp17.6C-CI | Heat shock protein 17.6C (class I) | *Arabidopsis thaliana* | Rabbit (polyclonal) | 1/1,000 | gift of Prof. Elisabeth Vierling (University of Massachusetts) |
| SHMT1 | Serine hydroxymethyltransferase 1 | *Spinacia oleracea* | Rabbit (polyclonal) | 1/5,000 | Agrisera (Vännäs, Sweden), AS05 075 |
| GDC-H | Glycine decarboxylase subunit H | *Spinacia oleracea* | Rabbit (polyclonal) | 1/5,000 | Agrisera (Vännäs, Sweden), AS05 074 |
| CCMA | ABC transporter I family member 1 | *Arabidopsis thaliana* | Rabbit (polyclonal) | 1/1,000 | Rayapuram *et al*., 2007 |
| CcmF_N1_ | Cytochrome *c* biogenesis CcmF N-terminal-like mitochondrial protein 1 | Ovalbumin-coupled peptide | Rabbit (polyclonal) | 1/20,000 | Rayapuram *et al*., 2008 |
| CcmF_N2_ | Cytochrome *c* biogenesis CcmF N-terminal-like mitochondrial protein 2 | Ovalbumin-coupled peptide | Rabbit (polyclonal) | 1/20,000 | Rayapuram *et al*., 2008 |

**References**

Lamattina, L.; Gonzalez, D.; Gualberto, J.; Grienenberger, J. M. Higher plant mitochondria encode an homologue of the nuclear-encoded 30-kDa subunit of bovine mitochondrial complex I. *Eur. J. Biochem*. **1993**, *217*, 831–838, DOI: [10.1111/j.1432-1033.1993.tb18311.x](http://onlinelibrary.wiley.com/doi/10.1111/j.1432-1033.1993.tb18311.x/abstract;jsessionid=A63EFD7E58BB23D6127A4525425411B1.f02t02).

Rayapuram, N.; Hagenmuller, J.; Grienenberger, J. M.; Bonnard, G.; Giegé, P. The Three Mitochondrial Encoded Ccmf Proteins Form a Complex That Interacts with CCMH and *c*-Type Apocytochromes in *Arabidopsis*. *J. Biol. Chem*. **2008**, *283*, 25200–25208, DOI: [10.1074/jbc.M802621200](http://www.jbc.org/content/283/37/25200.long).

Rayapuram, N.; Hagenmuller, J.; Grienenberger, J.-M.; Giegé, P.; Bonnard, G. AtCCMA Interacts with AtCcmB to Form a Novel Mitochondrial ABC Transporter Involved in Cytochrome *c* Maturation in *Arabidopsis*. *J. Biol. Chem*. **2007**, *282*, 21015–21023, DOI: [10.1074/jbc.M704091200](http://www.jbc.org/content/282/29/21015.long).
